# CPAP promotes angiogenesis and metastasis by enhancing STAT3 activity

**DOI:** 10.1038/s41418-019-0413-7

**Published:** 2019-09-11

**Authors:** Ruo-Yu Chen, Chia-Jui Yen, Yao-Wen Liu, Chun-Guo Guo, Chieh-Yu Weng, Chien-Hsien Lai, Ju-Ming Wang, Yih-Jyh Lin, Liang-Yi Hung

**Affiliations:** 1 Department of Biotechnology and Bioindustry Sciences, College of Bioscience and Biotechnology, National Cheng Kung University, Tainan, 70101 Taiwan; 2 Division of Hematology and Oncology, Department of Internal Medicine, National Cheng Kung University Hospital, College of Medicine, National Cheng Kung University, Tainan, 70101 Taiwan; 3 Department of Clinical Pathology, Kuo General Hospital, Tainan, 70054 Taiwan; 4 Institute of Bioinformatics and Biosignal Transduction, College of Bioscience and Biotechnology, National Cheng Kung University, Tainan, 70101 Taiwan; 5 Division of General and Transplantation Surgery, Department of Surgery, National Cheng Kung University Hospital, College of Medicine, National Cheng Kung University, Tainan, 70101 Taiwan; 6 Department of Pharmacology, College of Medicine, National Cheng Kung University, Tainan, 70101 Taiwan; 7 Ph. D. program for Cancer Molecular Biology and Drug Discovery, College of Medical Science and Technology, Taipei Medical University, Taipei, 11031 Taiwan; 8 Graduate Institute of Medicine, College of Medicine, Kaohsiung Medical University, Kaohsiung, 80708 Taiwan

**Keywords:** Metastasis, Tumour angiogenesis

## Abstract

Centrosomal P4.1-associated protein (CPAP) is overexpressed in hepatocellular carcinoma (HCC) and positively correlated with recurrence and vascular invasion. Here, we found that CPAP plays an important role in HCC malignancies. Functional characterization indicated that CPAP overexpression increases tumor growth, angiogenesis, and metastasis ex vivo and in vivo. In addition, overexpressed CPAP contributes to sorafenib resistance. Mechanical investigation showed that the expression level of CPAP is positively correlated with activated STAT3 in HCC. CPAP acts as a transcriptional coactivator of STAT3 by directly binding with STAT3. Interrupting the interaction between CPAP and STAT3 attenuates STAT3-mediated tumor growth and angiogenesis. Overexpression of CPAP upregulates several STAT3 target genes such as *IL-8* and *CD44* that are involved in angiogenesis, and *CPAP* mRNA expression is positively correlated with the levels of both mRNAs in HCC. Knocked-down expression of CPAP impairs IL-6-mediated STAT3 activation, target gene expression, cell migration, and invasion abilities. IL-6/STAT3-mediated angiogenesis is significantly increased by CPAP overexpression and can be blocked by decreased expression of *IL-8*. Our findings not only shed light on the importance of CPAP in HCC malignancies, but also provide potential therapeutic strategies for inhibiting the angiogenesis pathway and treating metastatic HCC.

## Introduction

Hepatocellular carcinoma (HCC) is the sixth most common malignancy worldwide and the second-leading cause of cancer-related mortality [1]. During HCC development, angiogenesis plays an important role in promoting tumor growth and metastasis, which leads to malignancy and poor prognoses [2, 3]. Angiogenesis is triggered by signals that are released from tumor cells; activation of signaling pathways that enhance angiogenesis as well as the production of angiogenic factors from cancer cells promotes angiogenesis [4–6]. Therefore, the development of antiangiogenic reagents can be regarded as a good strategy for HCC therapy [2, 3, 7, 8]. Sorafenib is one of the antiangiogenesis drugs approved by the FDA for metastatic HCC treatment [2]. Unfortunately, only a small group of patients respond to antiangiogenesis treatment, and the survival benefit is limited by severe side effects [3, 9]. According to the literature, several pathways, including PI3K/AKT, NF-κB, and STAT3, are involved in angiogenesis [7, 10, 11], and the activation of those pathways contributes to angiogenesis by increasing the expression of angiogenic factors, such as VEGF, IL-8, HIF1α, or IL-6 [11]. The effects of angiogenesis have also been well characterized; however, the mechanism of angiogenesis in HCC remains yet to be fully defined.

STAT3 is one important regulator involved in inflammation and carcinogenesis; activation of the IL-6/STAT3 pathway leads to the expression of many genes that regulate cell proliferation, anti-apoptosis, migration, angiogenesis, and malignant transformation during carcinogenesis [12]. Activated STAT3 is positively correlated with poor prognoses in HCC; nuclear tyrosine705-phosphorylated STAT3 (STAT3/Y705-p) was present in 60% of HCC and correlated with tumor progression and poor prognosis [12, 13]. Importantly, augmented STAT3 activity is closely correlated with increased histological grading and intratumoral microvessel density in HCC [13].

Centrosomal P4.1-associated protein (CPAP) was originally identified as a centrosomal protein that plays an important role in centrosome functions, including regulating centriole elongation and microtubule polymerization during cell division [14, 15]. CPAP not only plays an important role in centrosome function but also serves as a transcriptional coactivator of STAT5 and NF-κB [16, 17]. Our previous studies indicated that CPAP can be SUMO-1 modified upon TNF-α treatment and that SUMOylated CPAP can recruit additional IKK complexes to phosphorylate IκBα, leading to increased degradation of IκBα and enhancing the activation of NF-κB. CPAP is overexpressed in HCC, and the expression of CPAP is positively correlated with vascular invasion, recurrence after surgery, and cancer staging in HCC [17].

To investigate the role and underlying mechanism of CPAP in angiogenesis and metastasis in HCC, orthotopic and splenic injection animal models were performed, and it was found that CPAP could enhance tumor growth and metastasis in vivo. Interestingly, both ex vivo and in vivo experimental results indicated that CPAP overexpression promotes tumor growth, metastasis, and angiogenesis by enhancing STAT3 transcriptional activity via direct interactions. The interacting domains between CPAP and STAT3 were mapped. Blockage of the IL-6/STAT3 pathway attenuated CPAP-mediated angiogenesis and cell migration, suggesting that the effect of CPAP on angiogenesis is STAT3 dependent. Overexpression of CPAP increases the expression of several genes downstream of STAT3, such as *HIF1α*, *IL-8*, *MCAM*, and *CD44*. The expression of CPAP is not only parallel with activated STAT3 in human HCC, but also positively correlated with IL-8 secretion, and *CD44* mRNA overexpression. Knockdown of *IL-8* expression abolished CPAP-mediated cell migration and angiogenesis. Therefore, our studies indicated that CPAP overexpression correlates with HCC malignancies and can serve as a prognostic indicator for HCC patients.

## Materials and methods

### Human HCC samples

Human clinical HCC tissues were obtained from National Cheng Kung University Hospital. The use of clinical HCC specimens was in accordance with the Declaration of Helsinki. This study was approved by the Institutional Review Board (IRB) of National Cheng Kung University Hospital, Tainan, Taiwan (B-ER-104-245).

### Cell culture, transfection, TNF-α, IL-6 treatment, and collection of conditioned media

Hep3B and HepG2 cells were purchased from BCRC (Food Industry Research and Development Institute, Taipei, Taiwan). Huh7 cells were purchased from JCRB cell bank (Japanese Collection of Research, Bioresources Cell Bank, Osaka, Japan). These cells were certificated using Short Tandem Repeat genotyping by Center for Genomic Medicine, National Cheng Kung University; and tested the mycoplasma contamination once per month. Hep3G and HepG2 cells were cultured in Dulbecco’s Modified Eagle’s Medium (DMEM/high glucose, 1954626, Gibco, Carlsbad, CA, USA). Huh7 cells were maintained in DMEM (low glucose, 1732501, Gibco). Media were supplemented with 10% FBS (10437-028, Gibco), 100 μg/ml streptomycin, and 100 unit/ml penicillin (30-002, Corning, NY, USA). HCC cells stably expressing GFP, GFP-CPAP, and GFP-PN1 were maintained in medium containing 0.5 mg/ml G418 (A1720, Sigma-Aldrich). Plasmids were transfected into cells using PolyJet^™^ (SL100688, SignaGen, Rockville, MD, USA). siRNA transfection was performed using Lipofectamine 2000 according to the manufacturer’s instructions (11668-500, Invitrogen, Carlsbad, CA, USA). For IL-6 or TNF-α treatment, cells were starved for 18–20 h by serum-free medium and then treated with 25 ng/ml IL-6 [18] (GF338, Millipore, Billerica, MA, USA) or 10 ng/ml TNF-α [17, 19] (GF314, Millipore, Billerica, MA, USA) for various time periods as indicated in the text. After treatment, the conditioned media were collected, concentrated by Amicon® Ultra Filters (UFC903008, Millipore), and then stored at −80 °C before use.

### Xenograft subcutaneous injection, orthotopic injection, and intrasplenic injection

All animal studies were performed according to protocols approved by the Laboratory Animal Center, Medical College, National Cheng Kung University (Approval No. 105032). For xenograft subcutaneous injection, 2 × 10^6^ cells were mixed with Matrigel (354234, Corning, Tewksbury, MA) (1:1, v/v) in a total volume of 100 μl and then subcutaneously injected into 6-week-old NOD-SCID mice. Tumor size was measured every 4–5 days, and tumor volume was calculated using the formula length × width^2^  ×  0.5. Six-week-old male nude mice were used for orthotopic injection and splenic injection. For orthotopic injection, 2 × 10^6^ cells were mixed with Matrigel (1:1, v/v) in a total volume of 25 μl and injected into the left lobe of the liver. For intrasplenic injection, 1 × 10^6^ cells in a total volume of 25 μl were directly injected into the spleen. After the mice were sacrificed, the xenograft tumors, spleen, liver, or lung tissues were collected for analysis.

### Western blot analysis and immunoprecipitation assay

Total cell lysates were collected for Western blot analysis or coimmunoprecipitation assay as described [17]. Cell fractions were collected using Nuclear Extraction Kit (SK-0001, Signosis, Santa Clara, CA, USA) according to the manufacturer’s instructions. The antibodies used in this study are described below: GFP (JL-8, Clontech, Mountain View, CA, USA), STAT3 (9139, Cell signaling, Danvers, MA, USA), phospho-STAT3/Y705 (9145, Cell signaling), GAPDH (sc32233, Santa Cruz, Dallas, TX), and α-tubulin (DM1A, T6299, Sigma-Aldrich, St. Louis, MO, USA). The CPAP polyclonal antibody was the same as that used in our previous studies [17]. Protein signals were detected using secondary HRP-conjugated anti-mouse or anti-rabbit antibodies by the Western Lightning Plus-ECL system (PerkinElmer, Waltham, MA, USA).

### In vivo Matrigel plug assay

For the in vivo Matrigel plug assay, cells were pretreated with IL-6 (25 ng/ml) for 24 h, and then resuspended in serum-free medium containing 25 ng/ml IL-6 (5 × 10^6^ cells in 50 μl) and mixed with 250 μl of Matrigel. The cell/Matrigel mixture was subcutaneously injected into 5–6-week-old male nude mice. Seven days after inoculation, the mice were sacrificed, and the tumor plugs were excised for analysis. The blood vessels were observed by immunohistochemical (IHC) staining as previously described [20] using an anti-CD31 antibody (77699, Cell Signaling).

### In situ proximity ligation assay (PLA)

In situ PLA was performed as described in the previous report [17]. Briefly, cells were seeded on a sterile 12-mm coverslip, transfected with full-length GFP-CPAP, GFP-CPAP fragments or Myc-STAT3 fragments, and then fixed with 3.7% formaldehyde for 10 min. In situ PLA was performed according to the manufacturer’s instructions (Olink Bioscience, Uppsala, Sweden) using the antibodies as described in the text. The interaction of proteins was amplified as distinct bright-red spots and detected using a fluorescence microscope (Personal DV Applied Precision, Issaquah, WA).

### Tumor metastasis RT^2^ PCR array

Total RNAs from 25 ng/ml IL-6-treated GFP/Hep3B or GFP-CPAP/Hep3B stable cells were collected for detecting the metastasis-related gene expression using the Human Tumor Metastasis RT^2^ Profiler PCR Array (PAHS-028Z, Qiagen, Frederick, MD, USA) according to the manufacturer’s instruction. Briefly, the total RNAs were purified using a Quick-RNA^™^ MiniPrep kit (Zymo Research, Orange, CA, USA), and reverse transcribed by a First Strand Synthesis Kit (Qiagen). The qPCR reaction is: 95 °C for 10 min, then 40 cycles at 95 °C for 15 s and 60 °C for 1 min. Each array contained six housekeeping genes (ACTB, B2M, GAPDH, HPRT1, RPLP0, and HGDC) used for normalization of the sample data. The array data was normalized against the house keeping genes by calculating the Δ*Ct* for each gene of interest in the plate. The fold changes of gene expression and scatterplot were analyzed using the online reference database.

### RNA extraction, reverse transcription, and real-time RT-PCR

Total RNA was isolated by TRIsure^™^ reagent (BIO-38033, Bioline, London, UK) and reverse transcribed into cDNA by the High-Capacity cDNA Reverse Transcription Kit (4368813, Applied Biosystems, Foster City, CA, USA). Quantitative real-time PCR (qPCR) was performed by SYBR® Green Supermix (170-8882, BIO-RAD, Hercules, CA, USA), and TaqMan qPCR was performed by TaqMan® Universal Master Mix II (4366597, Applied Biosystems). The expression level of mRNAs was normalized to that of *actin*. Primer sequences used for qPCR are: *CPAP* forward, 5′-AGCCCTCGAGATCCTCATCCCT-3′ and reverse, 5′-TAGCATGTCTGCGGCGTCCC-3′; *IL-8* forward, 5′-GACAAGAGCCAGGAAGAAACC-3′ and reverse, 5′-CTTTAGCACTCCTTGGCAAAA-3′; *VEGF* forward, 5′-CTACCTCCACCATGCCAAGT-3′ and reverse, 5′-CCATGAACTTCACCACTTCGT-3′; *HIF-1α* forward, 5′-GGCGCGAACGACAAGAAAAA-3′ and reverse, 5′-GTGGCAACTGATGAGCAAGC-3′; *ICAM-1* forward, 5′-GGCCTCAGTCAGTGTGA-3′ and reverse, 5′-AACCCCATTCAGCGTCA-3′; *IL-6* forward, 5′-AGAGTAACATGTGTGAAAGCAG-3′ and reverse, 5′-TCAGGACTTTTGTACTCATCTG-3′; *Actin* forward, 5′-CTGGACT TCGAGCAAGAGATG-3′ and reverse, 5′-TGATGGAGTTGAAGGTAGTTTCG-3′. Primer sequences used for TaqMan qPCR are: *CD44*: forward, 5′-GCAGTTTGCATTGCAGTCAAC-3′, reverse, 5′-TCTGTCCTCCACAGCTCCATT-3′ and probe, 5′-TCGAAGAAGGTGTGGGCAGAAGAAA-3′; *CD31* forward, 5′-TCCAACAGCGAGAAGATTTCTG-3′, reverse, CCACTTCTGTGTATTCTACATCCATGT-3′ and probe, 5′-AACAGCCATTACGGTTAT-3′.

### Luciferase reporter gene assay

Cells were cotransfected with STAT3-driven, *IL-6* promoter, *IL-8* promoter, or *HIF-1α* promoter luciferase reporter constructs and GFP, GFP-CPAP, or GFP-PN1 by PolyJet^™^ (SL100688, SignaGen). The *Renilla* luciferase reporter plasmid, phRG-TK, was cotransfected into cells for transfection efficiency control. Luciferase activities were measured using the Dual-Luciferase Reporter kit (Promega, Madison, WI, USA) according to the manufacturer’s instructions.

### Migration and invasion assays

Cell migration or invasion assays were performed using a Transwell filter (BD Biosciences, NJ, USA) according to the manufacturer’s instructions. For the migration assay, cells were seeded in the upper chamber containing a noncoated membrane. For the invasion assay, filters were coated with 100 μl of Matrigel matrix (300 μg/ml) for 1 h at 37 °C. Culture medium containing 10% FBS or conditioned medium was added to the lower chambers, and the cells were plated in the upper chamber with FBS-free medium. After 48 h, the cells were fixed with methanol and nonmigrated or noninvaded cells were removed from the upper surface of the filter. The cells on the lower surface of the membrane were stained with 0.1% crystal violet. The numbers of migrated cells were counted under an optical microscope. Migrated cells were counted in five fields and an average number was presented as the mean ± SEM. Each experiment was repeated at least three times.

### HUVEC migration assay and tube formation assay

For HUVEC migration assay, 1 × 10^5^ HUVECs were plated in the upper chamber with FBS-free medium; HCC cells or conditioned media were placed in the bottom of a 24-well plate. After 6 h, the cells were fixed with methanol and nonmigrated or noninvaded cells were removed from the upper surface of the filter. The cells on the lower surface of the membrane were stained with 0.1% crystal violet, and the numbers of migrating cells were counted under an optical microscope. Migrated cells were counted in five fields, and presented as the mean ± SEM. Each experiment was repeated at least three times. For the tube formation assay, 4 × 10^5^ HUVECs were seeded in a 96-well plate coated with Matrigel. After 6 h, capillary morphogenesis was evaluated by inverted microscopy. The number of branches was counted in five randomly selected fields, and presented as the mean ± SEM. Each experiment was repeated at least three times.

### Sorafenib treatment and cell viability assay

Hep3B stable cells (5 × 10^3^ cells/well) were seeded in 96-well plate and cells were treated with 10 μM Sorafenib [21, 22]; cell viability was determined by CCK-8 (96992, Sigma-Aldrich) and Caspase-Glo 3/7 Assay kit (G8091, Promega) at indicated times according to the manufacturer’s instructions.

### Statistical analysis

Statistical differences were assessed between the experimental groups using an unpaired Student’s *t* test with a one-tailed distribution or two-way analysis of variance (ANOVA). *p < 0.05; **p < 0.01; and ***p < 0.001.

## Results

### Overexpression of CPAP accelerates HCC tumor growth and metastasis

**Fig. 1:**
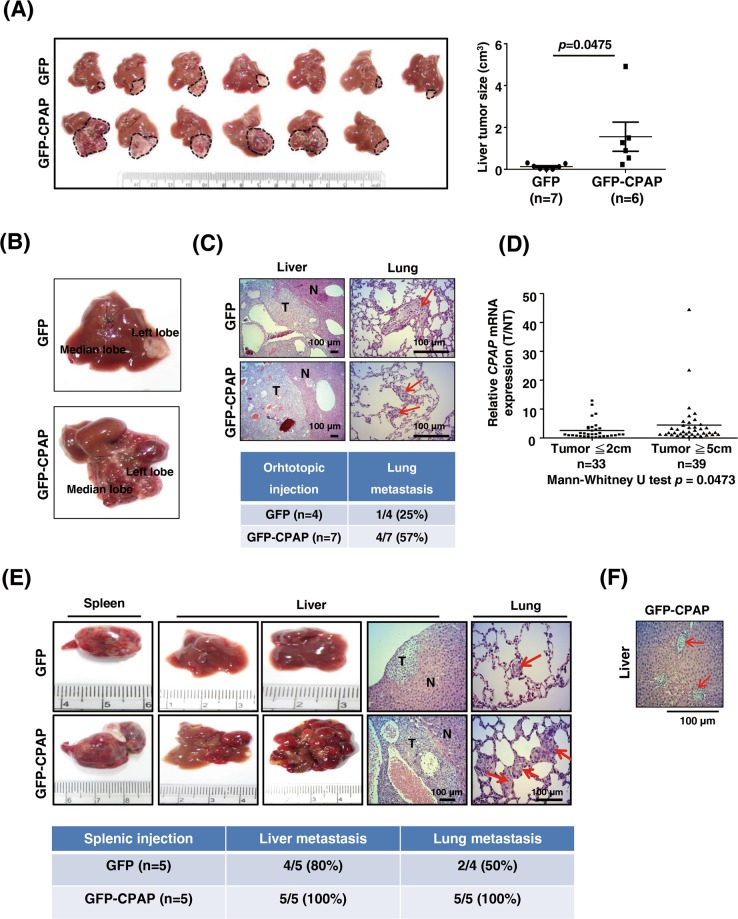
CPAP overexpression contributes to tumor growth and metastasis in vivo. **a** For orthotopic liver injection, GFP/Hep3B, or GFP-CPAP/Hep3B cells were injected into the left liver lobe of BALB/c nude mice. After 24 days of injection, the mice were sacrificed, and the liver tissues were collected for further analysis. Images of orthotopic liver xenograft tumors obtained from BALB/c nude mice injected with GFP/Hep3B (*n* = 7) or GFP-CPAP/Hep3B cells (*n* = 6) are shown (left); the tumor volume was calculated using the formula length × width^2^  ×  0.5. Quantitative results are shown as the mean ± SEM. Student’s *t* test, *p = 0.0475. **b** The liver tissues injected with GFP-CPAP/Hep3B but not GFP/Hep3B cells showed intrahepatic metastatic tumor growth. **c** After 8 weeks of injection, the mouse liver and lung tissues from orthotopically injected mice were collected for H&E staining. The incidence of lung metastasis is shown. **d** Seventy-two HCC specimens (T) and their adjacent normal tissues (NT) were collected to analyze the expression level of *CPAP* mRNA by RT-qPCR, which was presented as a ratio (T/NT). HCC tissues were divided into two size groups, small (≤2 cm, *n* = 33) and large (≥5 cm, *n* = 39). Mann–Whitney *U* test, *p* = 0.0473. RT-qPCR results were normalized to *actin*. **e** GFP/Hep3B (*n* = 5) or GFP-CPAP/Hep3B (*n* = 5) cells were intra-splenically injected into BALB/c nude mice. After 8 weeks of injection, the mice were sacrificed, and the spleen, liver, and lung tissues were collected for analysis. Representative images of the spleen and liver tumors, H&E staining of the liver and lung tissues, and the incidence of liver and lung metastasis are shown. **f** A representative image of H&E staining shows vascular invasion of the GFP-CPAP/Hep3B-derived liver tumor in the splenically injected mice. Arrows indicate vascular invasion of the GFP-CPAP/Hep3B cells. Scale bars, 100 μm

To investigate the effect of CPAP overexpression in hepatocarcinogenesis, we generated several HCC cells lines that stably expressed CPAP (Supplementary Fig. S1). We first investigated the growth-promoting ability of CPAP by performing orthotopic liver injection. As shown in Fig. 1a, after 24 days of injection, the tumor volume was enhanced in GFP-CPAP-overexpressing Hep3B cell (GFP-CPAP/Hep3B)-injected livers, and notable intrahepatic metastasis was observed in  GFP-CPAP/Hep3B-injected livers (Fig. 1b). These results implied that CPAP overexpression can promote HCC metastasis. As expected, after long-term follow-up ~8 weeks of injection, a high incidence of lung metastasis was observed in GFP-CPAP/Hep3B-injected mice (Fig. 1c). The frequency of lung metastasis was 57% (4/7) in GFP-CPAP/Hep3B-injected mice and only 25% (1/4) in GFP/Hep3B-injected mice (Fig. 1c). Interestingly, CPAP was overexpressed in HCC tissues with a large tumor diameter (≥5 cm), which possess higher metastatic ability [23, 24], rather than in tumors with a small size (≤2 cm) (Fig. 1d).

The promotion of metastasis by CPAP was further evaluated using a splenic injection mouse model. After 8 weeks of injection, the results showed that Hep3B cells stably expressing GFP or GFP-CPAP showed marked tumor formation in the spleen and induced liver metastasis at frequencies of 80% (4/5) and 100% (5/5), respectively (Fig. 1e). Mice injected with GFP-CPAP/Hep3B cells all exhibited lung metastasis (100%, 5/5), whereas only 50% (2/4) with the liver metastasis (4/5) of the GFP/Hep3B-injected mice exhibited lung metastasis (Fig. 1e). Marked vascular invasion was observed in GFP-CPAP/Hep3B-injected mouse livers (Fig. 1f). Interestingly, the expression level of *CPAP* mRNA was higher in the splenic tumors with lung metastasis found in GFP/Hep3B-injected mice (Supplementary Fig. S2). The increased CPAP expression in those mice might be due to individual differences.

**Fig. 2:**
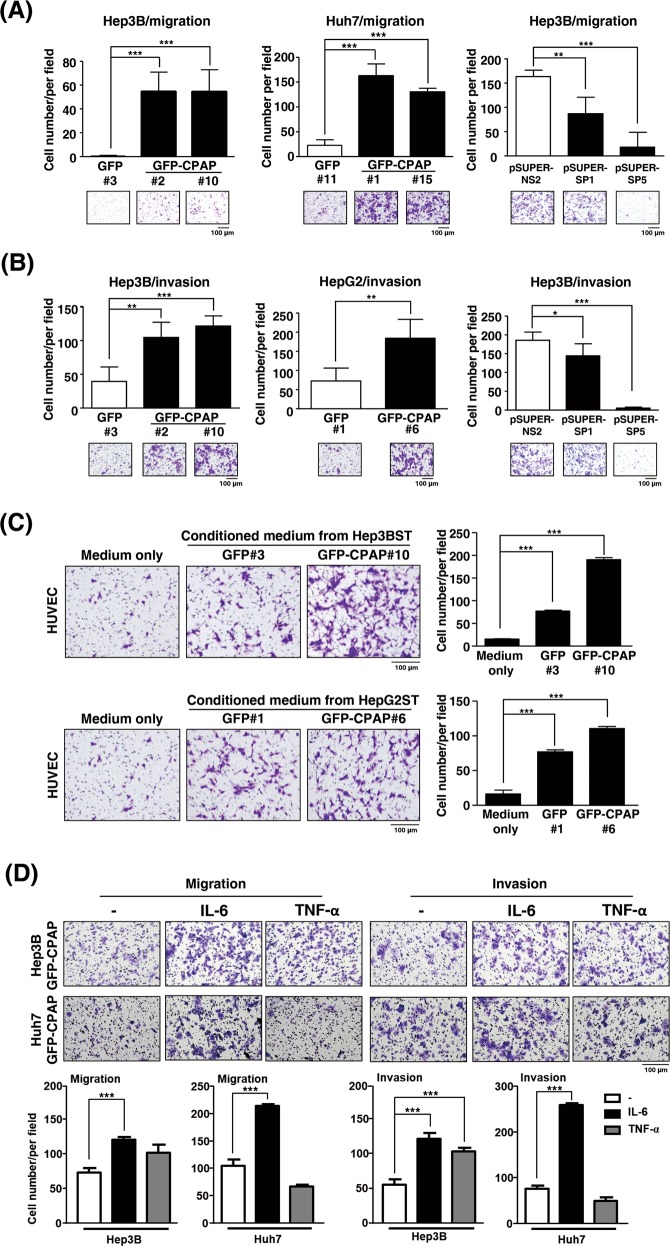
CPAP promotes cancer cell and HUVEC migration. **a**, **b** HCC cells stably expressing GFP, GFP-CPAP, or knocked-down CPAP by pSUPER-SP1 or pSUPER-SP5 were used to perform Transwell migration assays (**a**) or invasion assay (**b**). pSUPER-NS2 is a negative control of shRNA. **c** Conditioned media from IL-6-treated Hep3B (Hep3BST) and HepG2 (HepG2ST) cells stably expressing GFP or GFP-CPAP were collected to perform HUVEC migration assays. **d** Hep3B/GFP-CPAP or Huh7/GFP-CPAP stable cells were starved and treated without (–) or with IL-6 or TNF-α for 24 h, and then cell migration (left) and invasion (right) assays were performed. The number of migrated or invasive cells was counted, and the quantitative results are shown. Student’s *t* test, *p < 0.05, **p < 0.01, ***p < 0.001

To investigate the underlying mechanism involved in CPAP-enhanced metastasis, both Transwell migration and invasion assays were performed using CPAP-expressing stable cells (Supplementary Fig. S1) and CPAP-knocked-down cells [25] (Supplementary Fig. S3). The results showed that CPAP overexpression could enhance cell migration and invasion, whereas knockdown expression of CPAP decreases cell migration and invasion abilities (Fig. 2a, b). GFP or GFP-CPAP stable cells cultured in G418-containing medium present a lower cell migration and invasion abilities than the parental cells. We found that the morphology of GFP-CPAP/Hep3B cells was changed to a spindle shape with apparent filopodia (Supplementary Fig. S4A) and presented loosened cell–cell interactions (Supplementary Fig. S4B). It is well recognized that angiogenesis promotes cancer metastasis [2, 5]; therefore, we examined the angiogenesis-promoting ability by CPAP overexpression. The results from the HUVEC migration assay showed that conditioned medium collected from GFP-CPAP/Hep3B cells, but not GFP/Hep3B, could enhance HUVEC migration (Fig. 2c, upper panel). The same results were obtained in GFP-CPAP/HepG2 cells (Fig. 2c, lower panel).

To further dissect the signaling pathway(s) involved in CPAP-enhanced metastasis, HCC cells were treated with TNF-α or IL-6, both of which are reported to play a vital role in regulating cell migration and angiogenesis [11, 12, 26], to determine the cell migration and invasion abilities. The results indicated that IL-6 treatment significantly promoted GFP-CPAP-enhanced cell migration and invasion; the TNF-α effect is significant in cell invasion in Hep3B cells, but has no effect on CPAP-mediated cell migration (Fig. 2d).

### CPAP enhances IL-6/STAT3 pathway activity

**Fig. 3:**
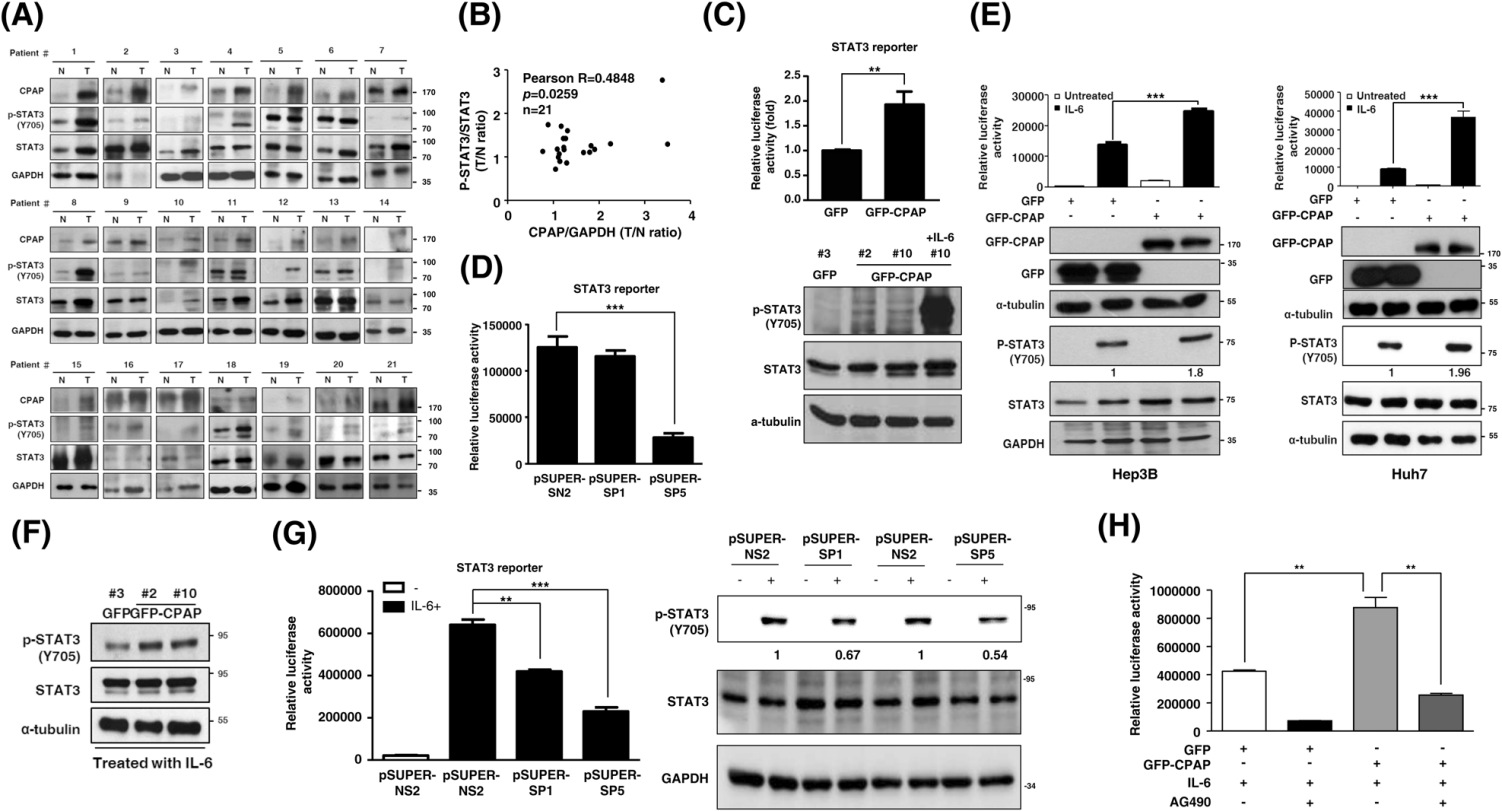
CPAP enhances STAT3 activity in HCC. **a, b** Immunoblotting analysis showed the expression of CPAP, p-STAT3, and STAT3 in paired HCC tumor (T) and adjacent nontumor tissues (N) (**a**). A total of twenty-one paired HCC specimens were analyzed. GAPDH was used as a loading control. A positive correlation between CPAP and p-STAT3 expression is shown (**b**). Pearson correlation coefficient, *R* = 0.4848, *p* = 0.0259. **c** (Upper) STAT3 transcriptional activity was determined in GFP- or GFP-CPAP-expressing Hep3B cells by a STAT3-driven luciferase reporter assay. STAT3 transcriptional activity is shown in relative luciferase activity as a fold change. (Lower) The level of phosphor-STAT3/Y705 (p-STAT3/Y705) in GFP/Hep3B or GFP-CPAP/Hep3B stable cells under normal culture conditions was determined by Western blot analysis. IL-6-treated GFP-CPAP/Hep3B cells were used as a positive control to detect p-STAT3/Y705. α-tubulin was used as a loading control. **d** STAT3 transcriptional activity in CPAP-knocked-down cells was determined by STAT3-driven luciferase reporter assay as described above. **e** GFP- or GFP-CPAP-expressing Hep3B or Huh7 cells were serum starved and then treated with (■) or without (□) IL-6 (25 ng/ml) for 6 h to determine the transcriptional activity of STAT3 by reporter assay as described above. Western blot analysis showed the expression of the indicated proteins. **f** Total lysates of GFP/Hep3B or GFP-CPAP/Hep3B stable cells treated with 25 ng/ml IL-6 for 10 min were collected for Western blot as described above. **g** CPAP-knocked-down Hep3B cells were treated with (■) or without (□) IL-6 to determine STAT3 activity by reporter assay (left) or Western blot analysis (right) as described above. **h** After serum starvation, GFP/Hep3B or GFP-CPAP/Hep3B cells were pretreated with (+) or without (−) 200 μM AG490 for 30 min and then treated with 25 ng/ml IL-6 for 6 h. STAT3 transcriptional activity was then determined by a STAT3-driven luciferase reporter assay. Student’s *t* test, *p < 0.05, **p < 0.01, ***p < 0.001

Next, we examined the expression and activation status of STAT3 in correlation with CPAP expression in clinical HCC tissues. The results showed that STAT3 is activated and positively correlated with CPAP overexpression in HCC (Fig. 3a, b). Using a STAT3-driven reporter assay and Western blot analysis, we found that CPAP overexpression can enhance the activation of STAT3 (Fig. 3c). Consistently, decreased expression of CPAP attenuates STAT3 transcriptional activity (Fig. 3d). Upon IL-6 treatment, STAT3 activity is largely enhanced in CPAP-overexpressing Hep3B and Huh7 cells (Fig. 3e, f), but not in CPAP-knocked-down cells (Fig. 3g). When cells were pretreated with AG490, a JAK2 inhibitor that blocks the activation of STAT3 (Supplementary Fig. S5), CPAP-enhanced IL-6/STAT3 activation was attenuated (Fig. 3h).

**Fig. 4:**
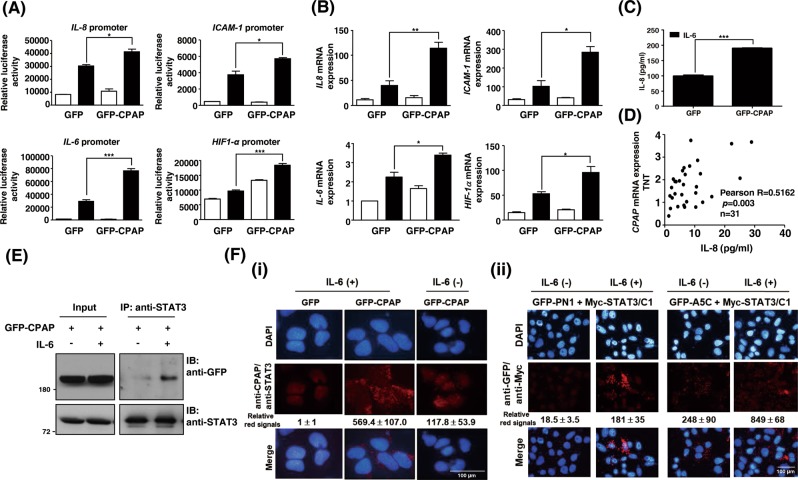
CPAP promotes the activity of the IL-6/STAT3 pathway. **a, b** The promoter activity (**a**) and gene expression level (**b**) of *IL-8*, *ICAM-1*, *IL-6*, and *HIF1-α* were determined in Hep3B cells expressing GFP or GFP-CPAP by reporter assay and RT-qPCR, respectively. Cells were serum starved, treated with (■) or without (□) IL-6 for 24 h, and then harvested for analysis. **c** GFP- or GFP-CPAP-expressing Hep3B cells were treated with IL-6 for 24 h; the medium was then replaced by fresh culture medium, and the cells were cultured for an additional 24 h. The conditioned media were collected to determine the expression of IL-8 by enzyme-linked immunosorbent assay (ELISA). **d** Sera from HCC patients (*n* = 31) were used to perform IL-8 ELISA. HCC tissues (T) and paired nontumorous tissues (NT) were used to analyze the *CPAP* mRNA expression level by RT-qPCR. The serum expression level of IL-8 is positively correlated with *CPAP* mRNA overexpression (T/NT) in HCC patients; Pearson correlation coefficient, *R* = 0.5162, *p* = 0.003. Student’s *t* test, *p < 0.05, **p < 0.01, ***p < 0.001. **e** GFP-CPAP-expressing Huh7 cells were treated with (+) or without (−) IL-6 for 4 min, and then total cell lysates were collected to perform a coimmunoprecipitation assay using anti-STAT3 antibodies. The immunoprecipitated complexes were subjected to Western blot analysis using the indicated antibodies. **f** (i) In vivo PLA using anti-STAT3 and anti-CPAP antibodies in IL-6-treated (+) or untreated (−) GFP- or GFP-CPAP-expressing Huh7 cells. (ii) Huh7 cells were cotransfected with Myc-STAT3/C1 and GFP-PN1 or GFP-A5C and then treated with (+) or without (−) IL-6 for 4 min. The interaction between PN1 or A5C and Myc-STAT3/C1 was detected by in situ PLA using anti-GFP and anti-Myc antibodies. The red signals indicate positive interactions. DAPI is a DNA-specific dye. The quantitative results of PLA red signals are shown

The expression of STAT3 downstream genes such as *IL-8*, *ICAM-1*, *IL-6*, and *HIF1α* was determined by promoter assay and RT-qPCR in GFP-CPAP-overexpressing or CPAP-knocked-down cells. The data showed that IL-6 treatment could induce the expression of these genes, and overexpression of GFP-CPAP further enhanced their expression (Fig. 4a, b). When cells knocked down the expression of CPAP, the level of STAT3 target genes was decreased (Supplementary Fig. S6). Enzyme-linked immunosorbent assay (ELISA) confirmed that the expression level of IL-8 was increased in GFP-CPAP/Hep3B cells upon IL-6 treatment (Fig. 4c), and the serum expression level of IL-8 was positively correlated with CPAP overexpression in HCC patients (Fig. 4d).

### CPAP enhances STAT3 activity by directly interacting with the STAT3 SH2 domain

A previous report indicated that CPAP can enhance STAT5 activation upon prolactin stimulation by forming a complex with STAT5, but not the STAT1 and STAT3, using the C-terminal domain [16]. The interaction between CPAP and STAT5 was demonstrated in 293T cells using the ectopic expression of Flag-CPAP and Myc-STAT5 [16]. Here, our results indicated that CPAP can enhance the activity of STAT3 upon IL-6 treatment; thus, we checked the possible interaction between CPAP and STAT3 under IL-6 treatment in HCC cells. Both co-immunoprecipitation (co-IP) assay and in situ PLA indicated that CPAP can directly interact with STAT3 upon IL-6 treatment (Fig. 4e, 4f–i). In order to show the specific protein binding of co-IP, normal mouse IgG was used as an isotype control antibody (Supplementary Fig. 7). The interacting domains of CPAP and STAT3 were further dissected by co-IP and in situ PLA using various deletion fragments of CPAP and STAT3. Full-length CPAP was divided into four fragments, PN1, CM, A5M2, and A5C, and sub-cloned to express GFP-fusion proteins (Supplementary Fig. S8A [14],); STAT3 was divided into three fragments containing the N-terminal domain plus coiled–coiled domain and DNA-binding domain (STAT3-N), the linker domain plus SH2 and transactivation (TA) domain (STAT3-C1), and SH2 domain plus TA domain (STAT3-C2) (Supplementary Fig. S8B). The results showed that the PN1 and A5C fragments of CPAP as well as the C1 and C2 fragments of STAT3 participated in the interaction (Supplementary Fig. S8C and D). The interaction between the STAT3-C1 fragment and PN1 or A5C in response to IL-6 treatment was further verified by in situ PLA. The data showed that IL-6 can stimulate the interaction between STAT3-C1 and PN1 or A5C (Fig. 4f-ii). In addition, WB analysis of cell fractions (Supplementary Fig. S9A) and nuclear co-IP assay (Supplementary Fig. S9B) further demonstrated that CPAP and STAT3 can form a complex and co-localize in the nucleus.

### CPAP overexpression promotes migration and angiogenesis via the IL-6/STAT3 pathway

**Fig. 5:**
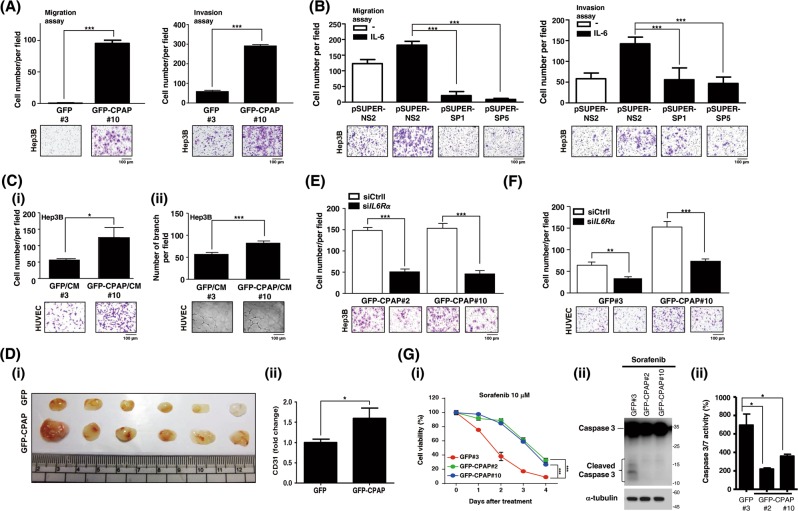
CPAP enhances angiogenesis via the IL-6/STAT3 pathway. **a**, **b** GFP/Hep3B, GFP-CPAP/Hep3B stable cells (**a**) or CPAP-knocked-down Hep3B cells (**b**) were treated with IL-6 for 24 h and then collected to perform Transwell migration and invasion assays. The number of migrated or invasive cells was counted, and the quantitative results are shown. **c** HUVEC Transwell migration assay (i) and tube formation assay (ii) were performed using conditioned medium (CM) collected from IL-6-treated Hep3B stable cells. The quantitative results of migration assay and tube formation assay are shown. **d** GFP/Hep3B or GFP-CPAP/Hep3B cells were used to perform Matrigel plug assay in NOD-SCID mice. The Matrigel plugs were excised (i) and analyzed by H&E staining and IHC assay using anti-CD31 antibodies (see Supplementary Fig. S9B). The IHC quantitative results are shown (ii). **e, f** GFP/Hep3B or GFP-CPAP/Hep3B cells transfected with si*IL-6*R were treated with IL-6 for 24 h and used in the cell migration assay (**e**) and HUVEC migration assay (**f**). Control siRNA (siCtrl) was used as a negative control. **g** GFP/Hep3B or GFP-CPAP/Hep3B stable cells were treated with 10 μM Sorafenib as indicated and the cell viability was determined by CCK-8. Two-way ANOVA analysis, ***p < 0.01 (i). Cells treated with 10 μM Sorafenib for 48 h were collected to check the apoptotic status by immunoblotting analysis using anti-caspase 3 antibodies (ii) and evaluating the caspase3/7 activity (iii). Student’s *t* test, *p < 0.05, **p < 0.01, ***p < 0.001

Next, we determined the role of IL-6 in CPAP-promoted cell metastasis. HCC cells stably expressing GFP, GFP-CPAP, or knocked-down CPAP were treated with IL-6, and then collected for performing the Transwell migration/invasion assay and HUVEC migration assay. The results showed notably increased cell migration, invasion (Fig. 5a, Supplementary Fig. S10A), and HUVEC migration (Supplementary Fig. S10B and C) in IL-6-treated GFP-CPAP-HCC stable cells. Hep3B cells with CPAP knocked-down expression are impaired in cell migration and invasion (Fig. 5b). Conditioned media collected from IL-6-treated GFP/Hep3B or GFP-CPAP/Hep3B cells were used to perform HUVEC migration and tube formation assays, and the results indicated that conditioned media from GFP-CPAP/Hep3B cells, but not GFP/Hep3B cells, can promote HUVEC migration and tube formation (Fig. 5c, Supplementary Fig. S11A). The promoting effect of CPAP on angiogenesis was further confirmed by an in vivo Matrigel plug assay. As Fig. 5d shows, GFP-CPAP/Hep3B cells induced more neovascularization than GFP/Hep3B cells (Fig. 5d–i). Both immunohistochemistry assay (Supplementary Fig. S11B-i, B-ii and Fig. 5d–ii) and RT-qPCR (Supplementary Fig. S11B-iii) showed a dense CD31 staining and enhanced *CD31* mRNA expression in GFP-CPAP/Hep3B-containing plug tumors compared with those containing GFP/Hep3B cells only, suggesting that GFP-CPAP can promote greater vessel formation. Importantly, in mice that received splenic injection (Fig. 1e), the expression level of *CD31* mRNA was higher in the GFP-CPAP/Hep3B-injected spleen tumors than in the GFP/Hep3B-injected tumors (Supplementary Fig. S12). Upon knocking down the expression of IL-6R by siRNA technology, the CPAP-enhanced HCC and HUVEC migrations were attenuated (Fig. 5e, f). Iin addition to promoting angiogenesis, CPAP-overexpressing HCC cells are more resistant to sorafenib, the antiangiogenesis drug, treatment (Fig. 5g); in contrast, knocked-down expression of CPAP enhances the cytotoxicity effect of sorafenib (Supplementary Fig. S13).

### The CPAP/PN1 fragment plays a dominant negative role in CPAP-enhanced STAT3 activation

**Fig. 6:**
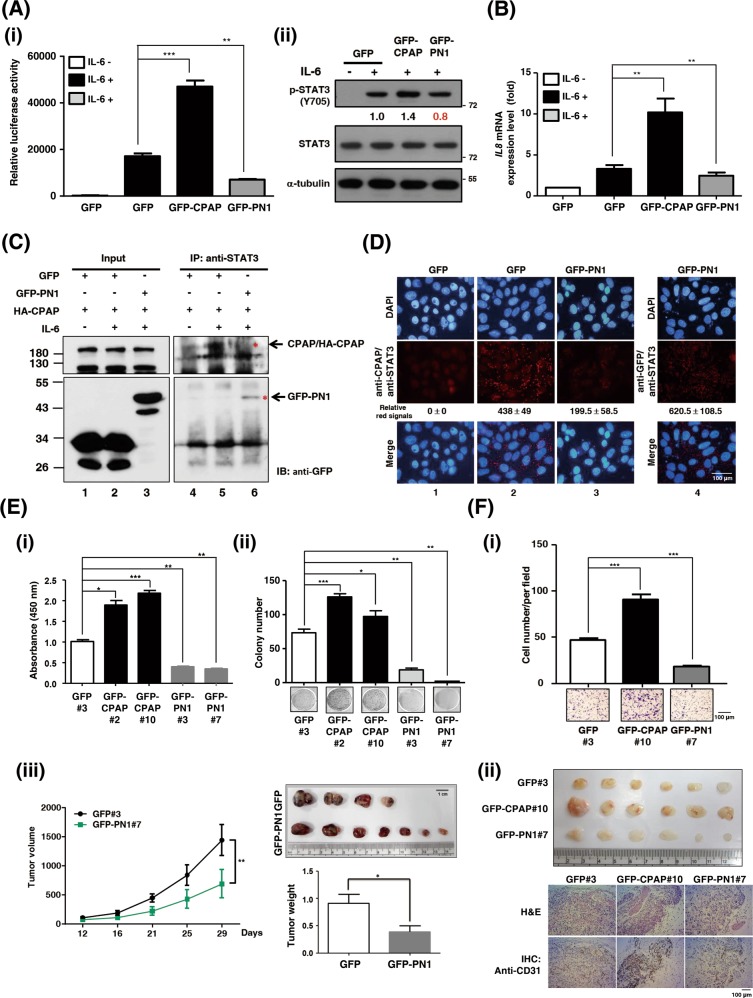
PN1 reduces cell proliferative, migrative, and angiogenic abilities by downregulating the IL-6/STAT3 pathway. **a, b** Hep3B cells transfected with GFP, GFP-CPAP, or GFP-PN1 were treated with IL-6 for the STAT3-driven reporter assay (**a**-i), Western blot analysis (**a**-ii), and RT-qPCR (**b**) as described above. **c** Huh7 cells were co-transfected with HA-CPAP and GFP or GFP-PN1; after serum starvation, cells were treated with (+) IL-6 and then used to perform the co-immunoprecipitation (co-IP) assay. **d** Huh7 cells transfected with GFP or GFP-PN1 were serum starvation (column 1), treated with IL-6 (columns 2–4), and then used to perform the in situ PLA using anti-STAT3 and anti-CPAP (columns 1–3) or anti-GFP (column 4) antibodies. The red signals show the interaction between STAT3 and CPAP or GFP-PN1. **e** Hep3B cells stably expressing GFP, GFP-CPAP, or GFP-PN1 were subjected to BrdU incorporation (i), colony formation (ii). (iii) GFP/Hep3B or GFP-PN1/Hep3B cells were subcutaneously injected into NOD-SCID mice. Tumor size was measured, and tumor volume was calculated. The quantitative results of the tumor weights are shown. Two-way ANOVA analysis, **p < 0.01. **f** After IL-6 treatment, GFP/Hep3B, GFP-CPAP/Hep3B, or GFP-PN1/Hep3B cells were collected for HUVEC migration (i) and Matrigel plug (ii) assays as described above. The Matrigel plugs were collected to perform H&E staining and an IHC analysis using the anti-CD31 antibody. Student’s *t* test, *p < 0.05, **p < 0.01, ***p < 0.001

To identify the functional domain of CPAP that contributes to IL-6/STAT3 activation, a STAT3-driven reporter assay was performed using cells transfected with CPAP/PN1 or CPAP/A5C fragments, both of which can interact with STAT3 (Figs. S8A and 4f-ii). Surprisingly, the IL-6-mediated STAT3 activity was diminished in GFP-PN1-overexpressing cells (Supplementary Fig. S14A, Fig. 6a–i), and GFP-PN1 or GFP-A5C has no effect on TNFα-induced NF-κB activation (Supplementary Fig. S14B). Western blot analysis showed that overexpression of GFP-PN1 decreases IL-6-induced STAT3 activation compared with that in GFP-expressing cells (Fig. 6a-ii). A similar effect was observed by analyzing the expression of STAT3 target genes such as *IL-8* (Fig. 6b).

Because our studies showed that CPAP/PN1 and A5C fragments interact with the STAT3 SH2 domain upon IL-6 treatment (Fig. 4f-ii), we hypothesized that overexpression of GFP-PN1 may compete with CPAP for interacting with STAT3. To test this hypothesis, co-IP assay and in situ PLA were performed. The results showed that HA-CPAP can interact with STAT3 upon IL-6 treatment as described above (Fig. 6c, lane 5). When cells were co-expressed with GFP-PN1, a complex was formed between GFP-PN1 and STAT3, and the interaction between HA-CPAP and STAT3 was decreased (Fig. 6c, lane 6). It indicated that PN1 can compete with CPAP for interacting with STAT3. The results from in situ PLA support this suggestion. Overexpression of GFP-PN1, but not GFP, leads to its interaction with endogenous STAT3 (Fig. 6d, compare columns 4 and 1); GFP-PN1, but not GFP, blocked the interaction between endogenous CPAP and STAT3 (Fig. 6d, compare columns 2 and 3). Cell proliferation, tumor growth, and angiogenesis were all reduced in GFP-PN1-expressing cells (Fig. 6e, f).

### IL6-induced *IL-8* and *CD44* expression mediates CPAP-promoted metastasis in HCC

**Fig. 7:**
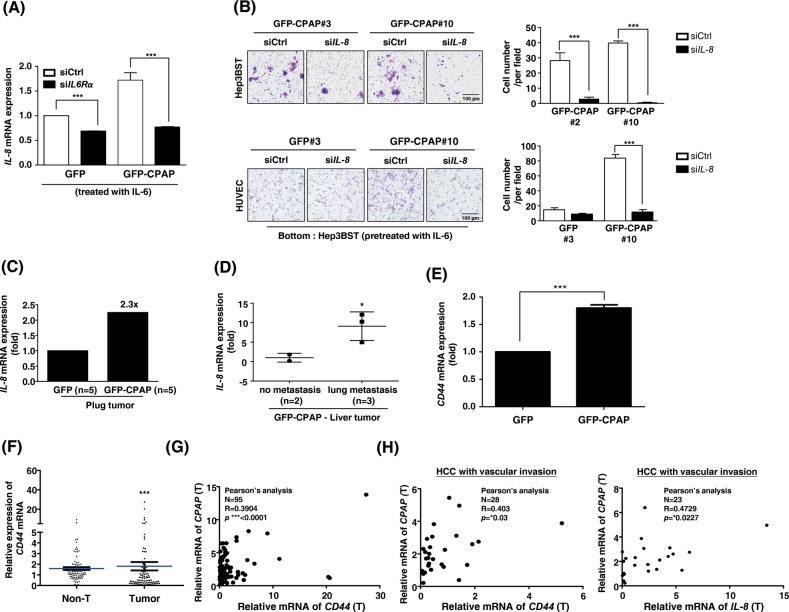
Several target genes of the IL-6/STAT3 pathway mediate CPAP-enhanced metastasis. **a** Expression of *IL-8* mRNA was determined in Hep3B cells cotransfected with siCtrl or si*IL-6R* and GFP or GFP-CPAP. **b** GFP/Hep3B or GFP-CPAP/Hep3B stable cells (Hep3BST) were transfected with si*IL-8* or siCtrl, treated with IL-6, and then used in the cell migration (upper) and HUVEC migration (lower) assays. Migrated cells were counted, and the quantitative results are shown. **c** The GFP- or GFP-CPAP-derived plug tumors (from Fig. 5d, *n* = 5 for either group) were pulled together for RNA purification and determined the expression level of *IL-8* mRNA by RT-qPCR. **d** Expression of *IL-8* mRNA was determined in GFP-CPAP-derived liver tumors with (*n* = 3) or without (*n* = 2) lung metastasis (from Fig. 1a) by RT-qPCR. **e** *CD44* mRNA level was determined by RT-qPCR in GFP/Hep3B and GFP-CPAP/Hep3B cells. **f, g** Expression of *CD44* mRNA in HCC tumor tissues and paired nontumor (Non-T) tissues was determined by RT-qPCR (**f**). Student’s *t* test, *p < 0.05, **p < 0.01, ***p < 0.001. A total of 95 HCC paired specimens were collected for analysis. The expression of *CD44* mRNA is positively correlated with *CPAP* mRNA in HCC tumor tissues (**g**). Pearson correlation coefficient, *R* = 0.3904, *p* < 0.0001. **h** The expression of *CD44* mRNA (left, Pearson correlation coefficient, *R* = 0.403, *p* < 0.03.) and *IL-8* mRNA (right, Pearson correlation coefficient, *R* = 0.4729, *p* < 0.0227.) is positively correlated with *CPAP* mRNA in HCC tumor tissues with vascular invasion. A total of 28 HCC specimens with vascular invasion were collected for analysis

According to the literature, IL-8 is an important regulator of angiogenesis in many cancer types as it directly enhances endothelial cell proliferation, survival, and MMP expression [27–29]. Our results showed that IL-8 expression is increased in CPAP-overexpressing HCC cells (Fig. 4a, b). Here, we investigated the effect of IL-8 on CPAP-enhanced IL-6/STAT3-mediated metastasis. As shown in our previous results, CPAP overexpression increased the expression of *IL-8* in the presence of IL-6; however, *IL-6R* siRNA impaired CPAP-enhanced IL-8 expression (Fig. 7a), suggesting an important role for CPAP in enhancing the expression of IL-8 by the IL-6/STAT3 pathway. HCC cell migration and HUVEC migration abilities decreased in *IL-8* siRNA-transfected GFP-CPAP/Hep3B cells (Fig. 7b). Plug tumors from the in vivo Matrigel plug assay (Fig. 5d) were used to evaluate the *IL-8* mRNA expression level. The data showed that *IL-8* expression is increased in GFP-CPAP/Hep3B-containing plug tumors compared with GFP/Hep3B-containing plug tumors (Fig. 7c). Liver tumors from the orthotopic model also showed similar results. The expression of *IL-8* mRNA was higher in GFP-CPAP-derived liver tumors (Fig. 1a) with lung metastasis than in non-metastasis liver tumors (Fig. 7d).

To identify additional molecules potentially involved in CPAP-mediated metastasis, we analyzed the gene expression pattern in the IL-6-treated GFP-CPAP/Hep3B cells using the human tumor metastasis RT^2^ Profiler^™^ PCR array. Only genes with expression level increase greater than two-fold were of interest; *CD44* is one gene that was upregulated in IL-6-treated GFP-CPAP/Hep3B cells (Supplementary Fig. S15). Because CD44 has been reported to be involved in HCC progression [30], we further confirmed their expression level and correlation with CPAP in HCC. As shown in Fig. 7e, *CD44* mRNA expression was increased in GFP-CPAP/Hep3B cells. The level of *CD44* mRNA was increased in the tumor parts rather than the adjacent nontumor tissues (Fig. 7f), and positively correlated with the level of *CPAP* mRNA in HCC (Fig. 7g). Importantly, the level of *CPAP* mRNA was positively associated with the expression of *CD44* and *IL-8* mRNAs in tumor tissue with vascular invasion (Fig. 7h).

## Discussion

Inhibition of the IL-6/STAT3 pathway is a promising approach for cancer treatment; however, STAT3 inhibitors that can be used at present in clinical cancer therapy are limited in their efficiency, specificity, and safety [31]. In this study, we found that CPAP can increase STAT3 activity by directly interacting with STAT3. This is the first report to demonstrate the interaction between CPAP and STAT3, as well as the first study to show the transcriptional coactivation effect of CPAP on STAT3 activity.

A previous report indicated that CPAP associates with STAT5a and STAT5b, but not STAT1 and STAT3 [16]. This is not the same condition as this study. In that report, the interaction between CPAP and STAT5 was demonstrated in 293T cells with ectopic expressed Flag-CPAP and Myc-STAT5, either in the presence or in the absence of constitutive activated Jak2, a known activator of STAT proteins [16]. No interaction was observed between CPAP C-terminal and STAT1 or STAT3 in 293T cells regardless of the presence of constitutive activated Jak2 [16]. In this study, our data showed that CPAP directly interacts with STAT3 upon IL-6 treatment in HCC cells (Fig. 4e, 4f-i), and the interacting domains were mapped (Supplementary Fig. S8C and D). Since STAT1, STAT3, and STAT5 have been reported as oncoproteins [32, 33], their roles in HCC development may differ. It was reported that the activation of STAT5 acts as a tumor suppressor via controlling the expression of ROS-induced NOX4 and two proapoptotic proteins, PUMA and BIM, in the liver tissue [34]. Deletion of STAT5 in the hepatocyte induces liver fibrosis and hepatocarcinogenesis through promoting the activation of STAT3 [35]. Since the activation of STAT3 is one of the major pathways in regulating the malignant features of HCC [11–13, 26], there is no doubt that CPAP-enhanced HCC metastasis occurs through the increased activation of the IL-6/STAT3 pathway.

Our results show that CPAP-overexpressing HCC cells exhibit enhanced tumor growth and metastatic abilities in an orthotopic animal model (Fig. 1). Inhibition of STAT3 activation by specific inhibitors, such as AG490, or knockdown of IL-6R nearly abolishes the effect of CPAP on HCC maligancy. Although our data suggested that the effect of CPAP is based on the activation of STAT3, we found that intefering with the interaction between CPAP and STAT3 by the CPAP/PN1 fragment largely abolished STAT3 activation in HCC cells (Fig. 6). Our study not only addresses the functional role of CPAP in STAT3-mediated hepatocarcinogenesis, but also provides a possibility for developing a potential peptide to block the CPAP-STAT3 interaction in CPAP-overexpressing HCC cells in the future.

IL-8 is known to possess tumorigenic, proangiogenic, and inflammatory properties [36]. IL-8 can be produced by endothelial cells [27], and other studies have indicated that tumor cells can also produce IL-8 to promote tumor growth, tissue invasion and distant metastasis [28, 37, 38]. A previous report indicated that the serum level of IL-8 is associated with tumor size and staging in HCC patients [39]; another study indicated that IL-8 expression in HCC tissues is not significantly correlated with the microvessel count, but is significantly correlated with the incidence of vessel invasion [37]. In this study, we found that CPAP overexpression increases IL-8 secretion in HCC cells (Fig. 4c) and that CPAP overexpression enhances angiogenic ability by IL-6/STAT3/IL-8 signaling activation (Fig. 7a, b). These results imply that CPAP is an important regulator of IL-8 signaling-mediated angiogenesis in HCC cells. Although, in our unpublished results, we did not link the expression level of *IL-8* mRNA with vascular invasion in clinical HCC tissues, a positive correlation between *CPAP* mRNA overexpression and *IL-8* mRNA in HCC tissues with vascular invasion was observed. The effect of CPAP on the crosstalk between tumor cells and endothelial cells remains to be clarified.

In addition to IL-8, CPAP overexpression also increases the expression of metastasis-related genes, including *CD44* (Supplementary Fig. S15, Fig. 7e). CD44 is a cancer stem cell marker that participates in a diverse set of functions in tumor cells, inculding cell proliferation, cell-to-cell adhesion, migration, invasion, and blood vessel development [40]. According to the literature, CD44 has prognostic value in HCC [30]. The expression level of CD44 correlates with vascular invasion and poor prognosis in HCC [30]. Our data showed that *CPAP* mRNA expression is positively correlated with *CD44* mRNA in HCC tissues with vascular invasion (Fig. 7h). These results suggest that CPAP is not only involved in metastasis but also contributes to other malignant properties to promote cancer progression in HCC.

There are many questions need to be further clarified. Our results showed that CPAP enhances the transcriptional activity of STAT3 and therefore increases the expression of STAT3-targeting genes; however, whether CPAP and activated STAT3 cotranslocates into the nucleus to bind to the promoter regions of target genes remains to be further clarified. In addition, the crosstalk between CPAP-regulated NF-κB and STAT3 activation in HCC remains unclear. Our previous report indicated that CPAP increases the transcriptional activity of NF-κB [17]; in this report, we demonstrated that CPAP enhances the activation of the IL-6/STAT3 pathway. Both NF-κB and STAT3 are important cancer-related inflammatory regulators [11], and they can interact with each other to enhance the activation of the inflammatory pathway and regulate the expression of the set of target genes. Although it was reported that there is crosstalk between STAT3 and NF-κB in regulating the inflammatory pathway and cancer development, the outcomes of this crosstalk are contradictory [11, 41]. Even though the role of CPAP in coordinating these two pathways remains unclear, our results show that CPAP might be an important regulator linking inflammation to cancer development. In summary, our findings indicate that CPAP is an important regulator of STAT3-mediated angiogenesis and cancer metastasis; hence, blockage of the interaction between CPAP and STAT3 is a promising strategy for cancer therapy in CPAP-overexpressing HCC cells.

## Supplementary information

Supplementary Figures

Supplementary Figures Legends
