# Supplementary material for: CPAP promotes angiogenesis and metastasis by enhancing STAT3 activity
Source: Cell Death Differ. 2019 Sep 11;27(4):1259–73. doi: 10.1038/s41418-019-0413-7 (PMC7206147; doi:10.1038/s41418-019-0413-7)
Supplement: Supplementary file 1 — Supplementary Figures [file 41418_2019_413_MOESM1_ESM.pdf]

Supplementary Figure S1

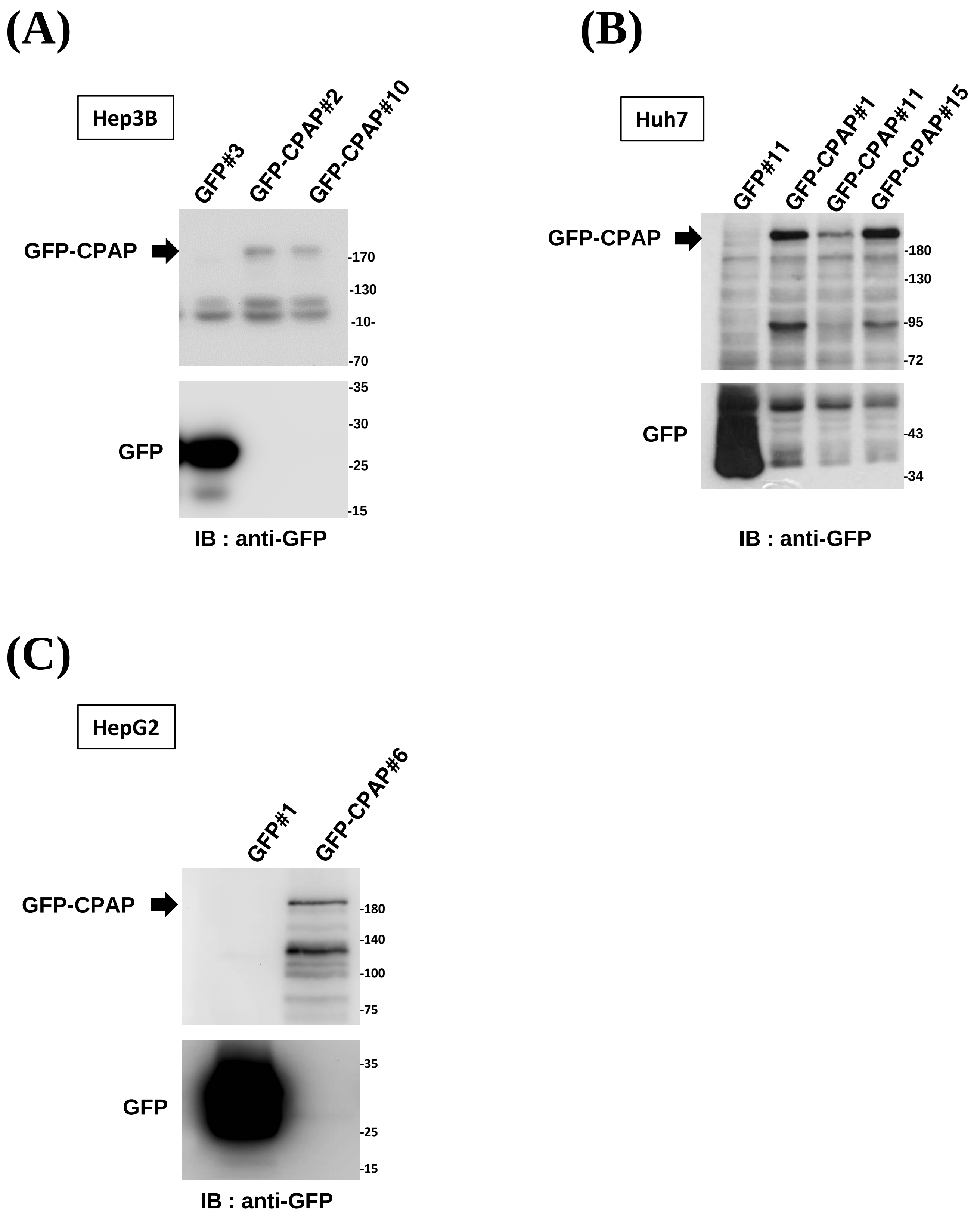

Supplementary Figure S2

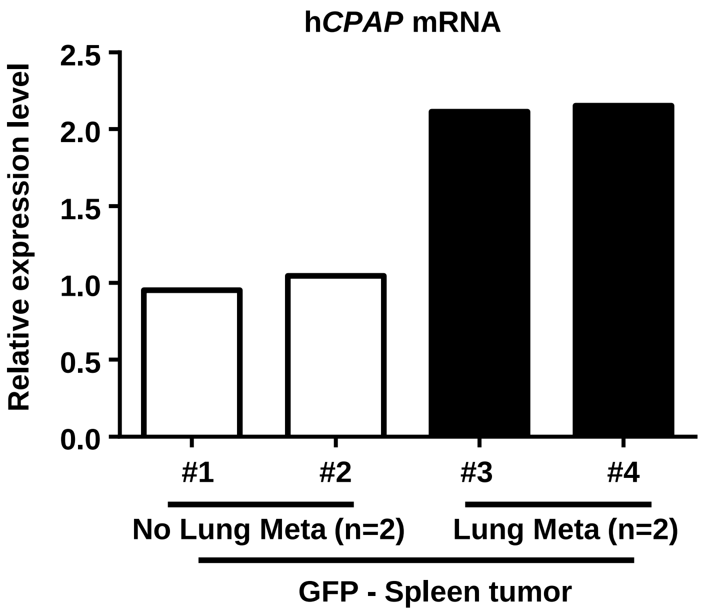

Supplementary Figure S3

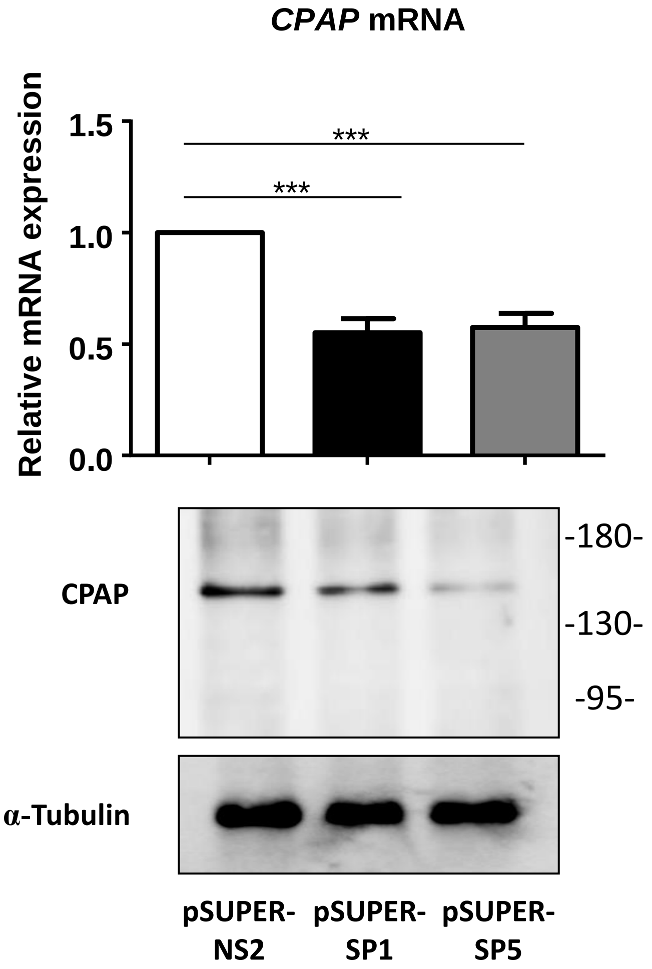

Supplementary Figure S4

(A)

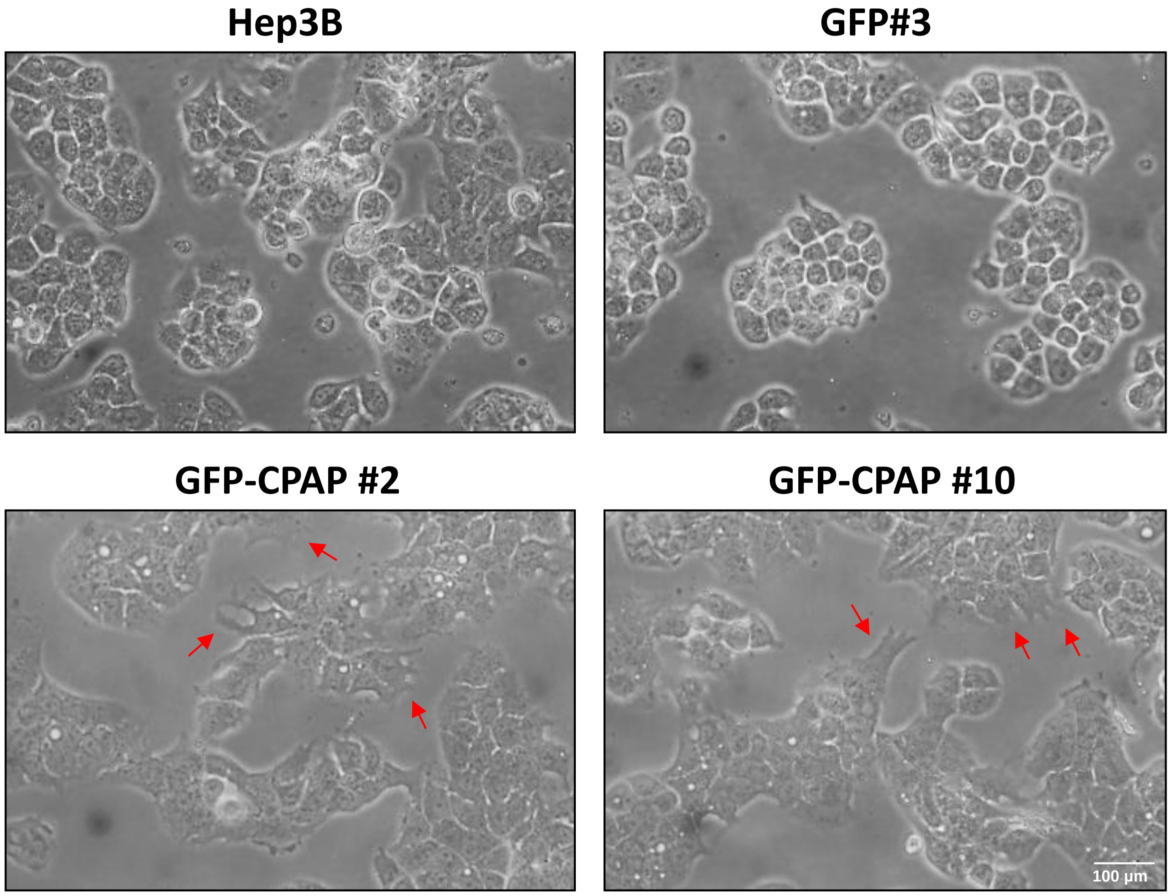

(B)

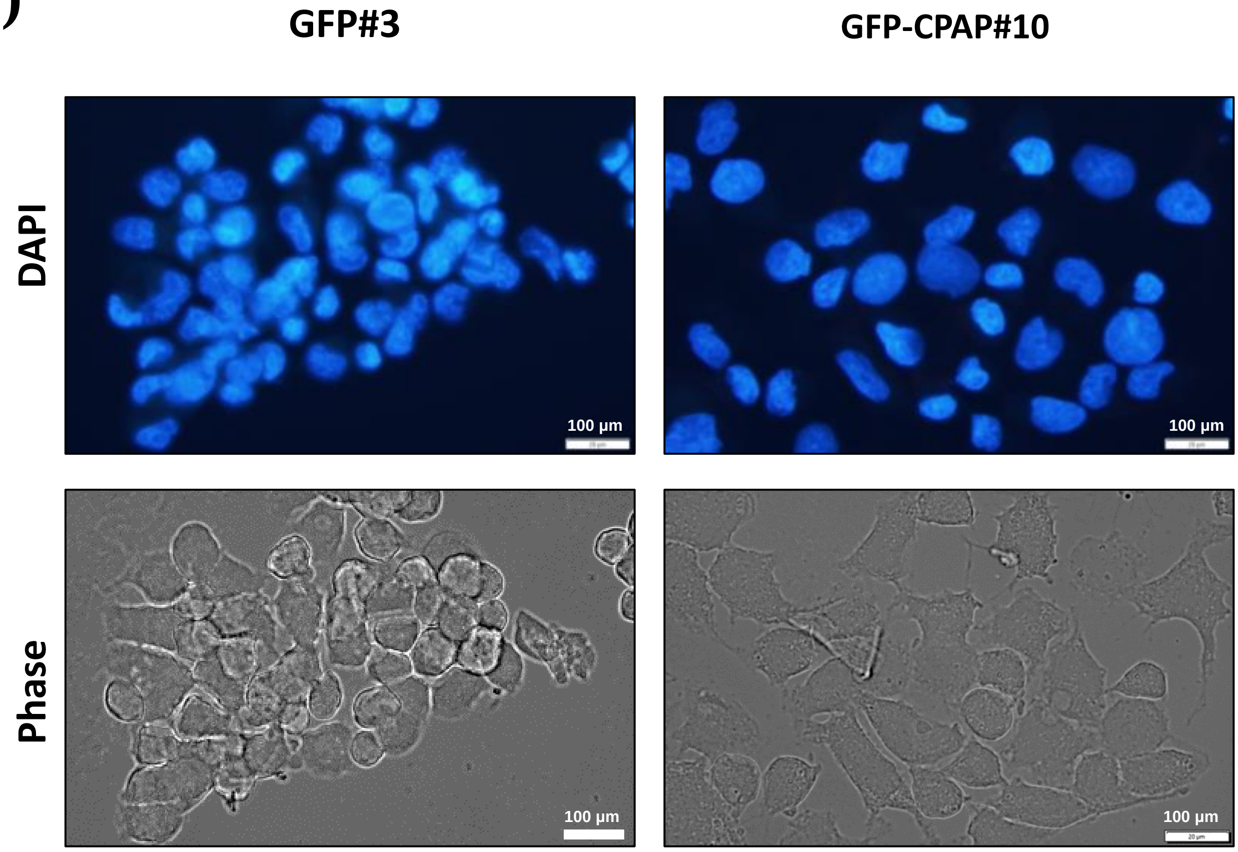

Supplementary Figure S5

(A)

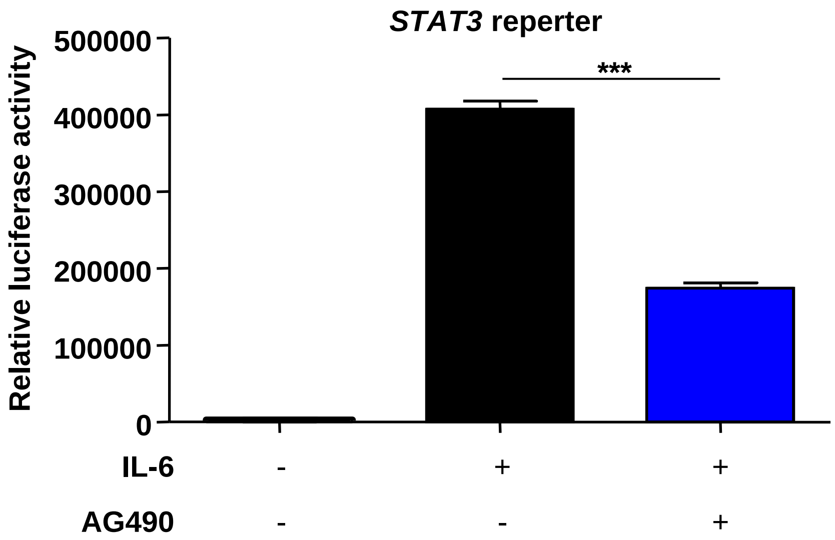

(B)

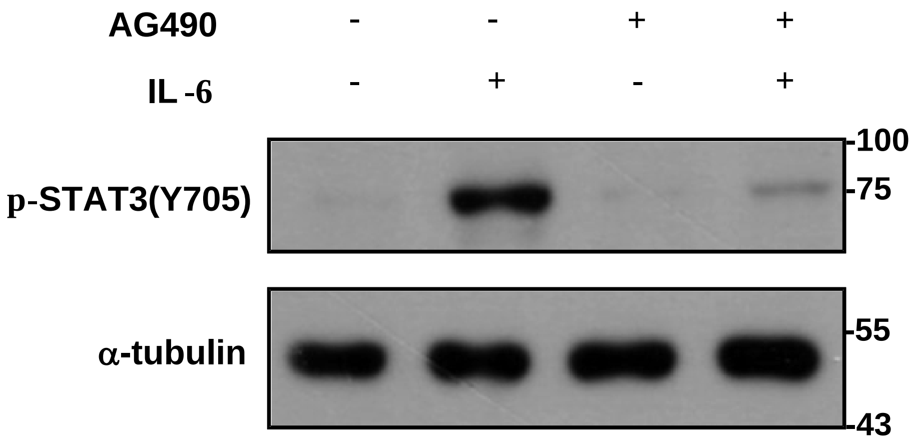

Supplementary Figure S6

(A)

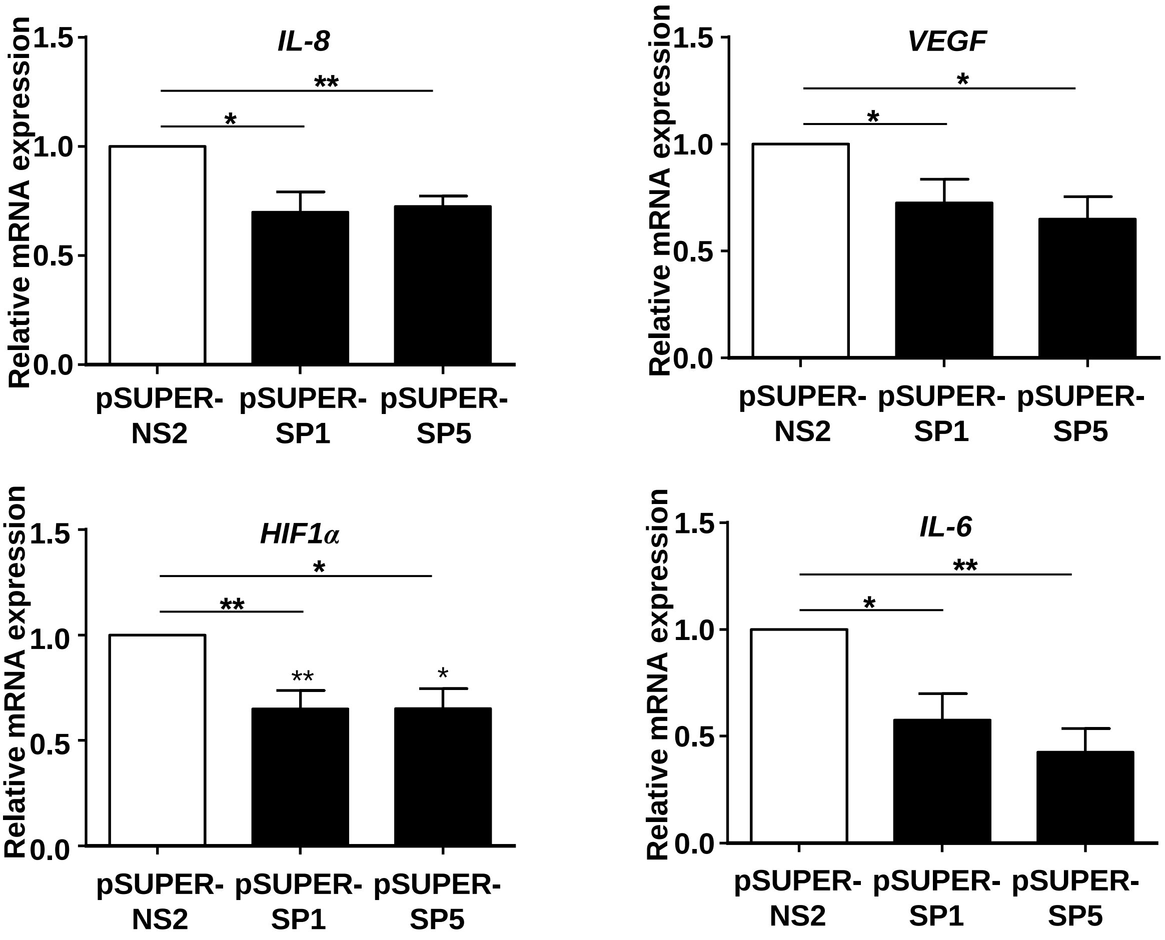

(B)

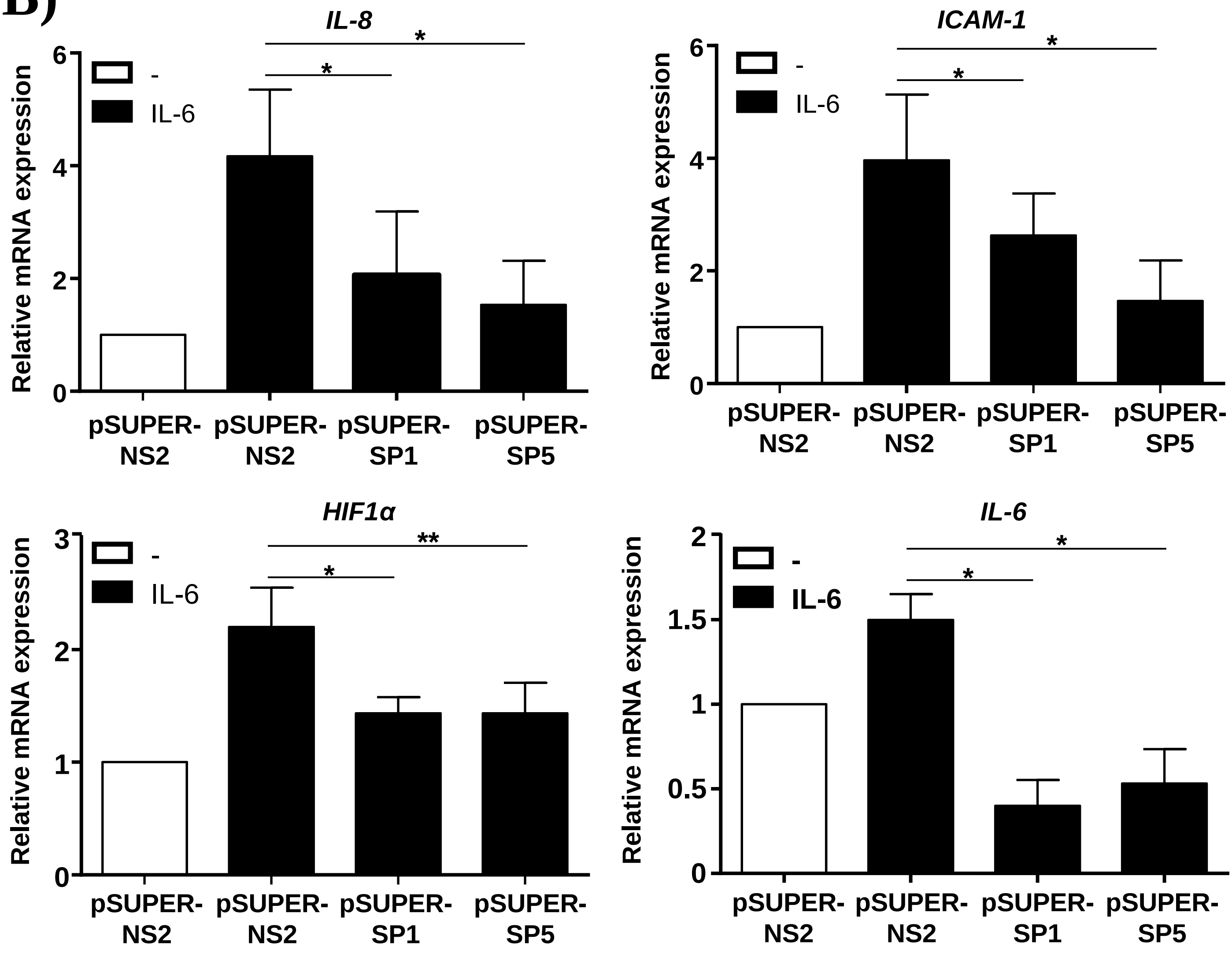

Supplementary Figure S7

(A)

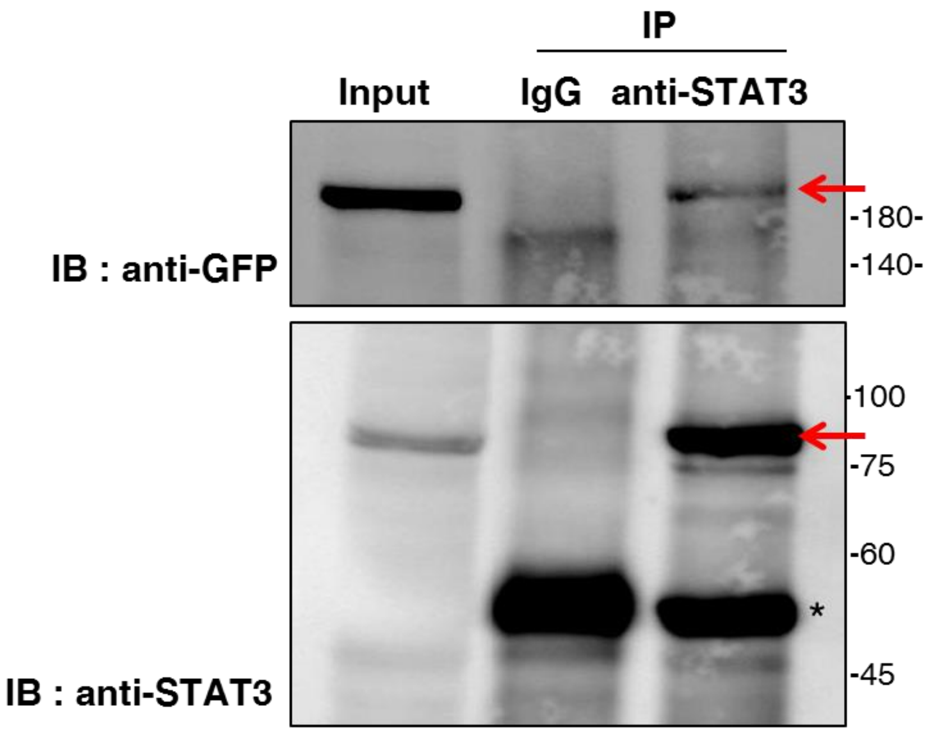

(B)

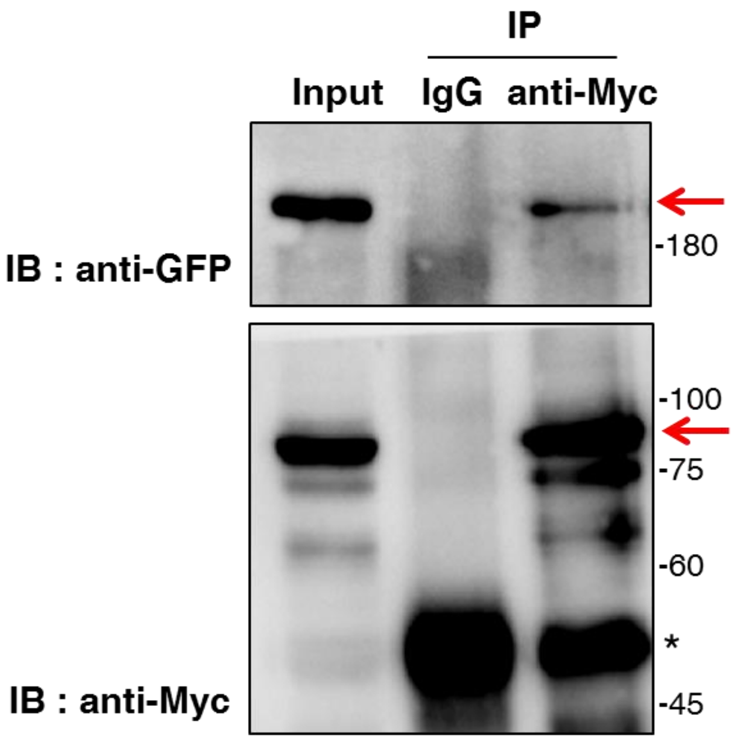

Supplementary Figure S8

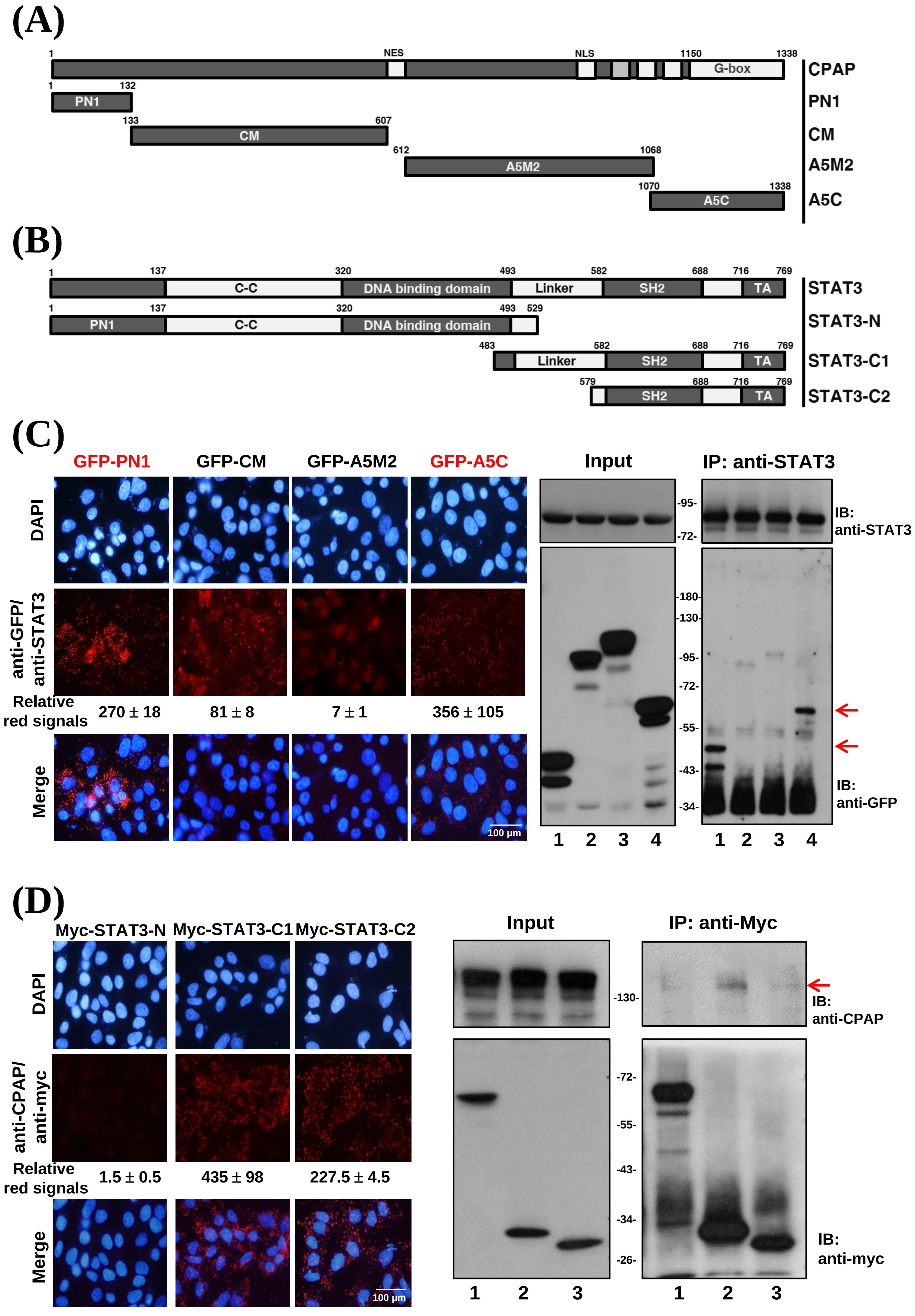

Supplementary Figure S9

(A)

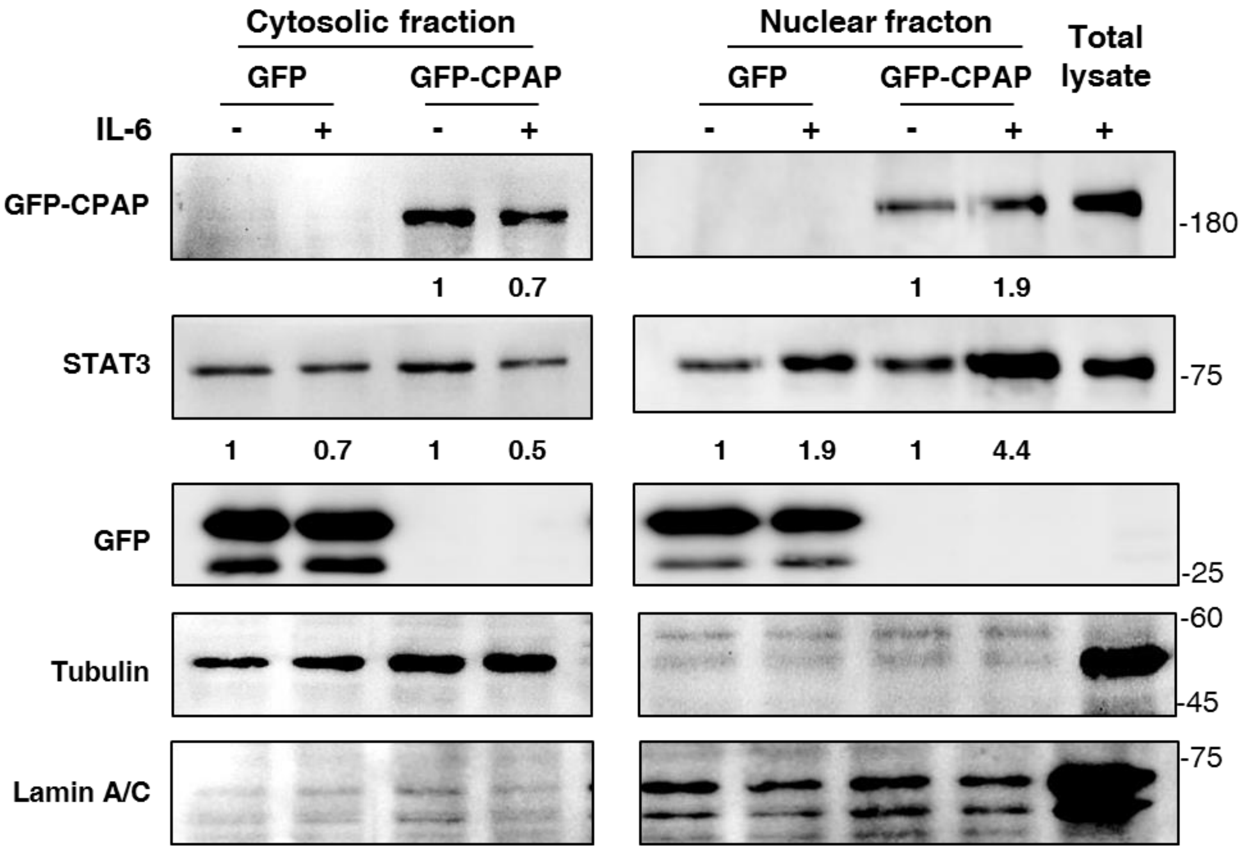

(B)

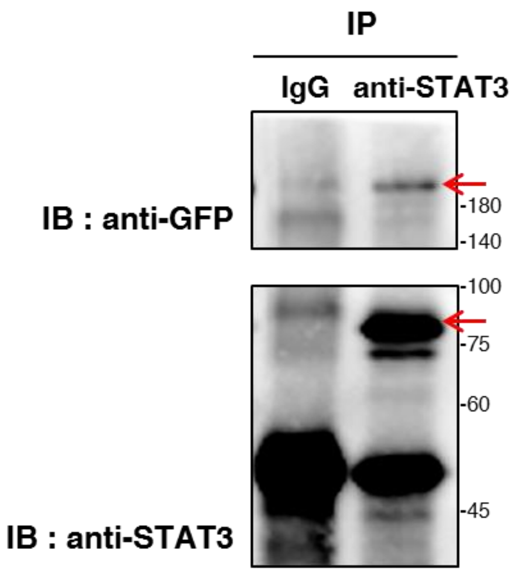

Supplementary Figure S10

(A)

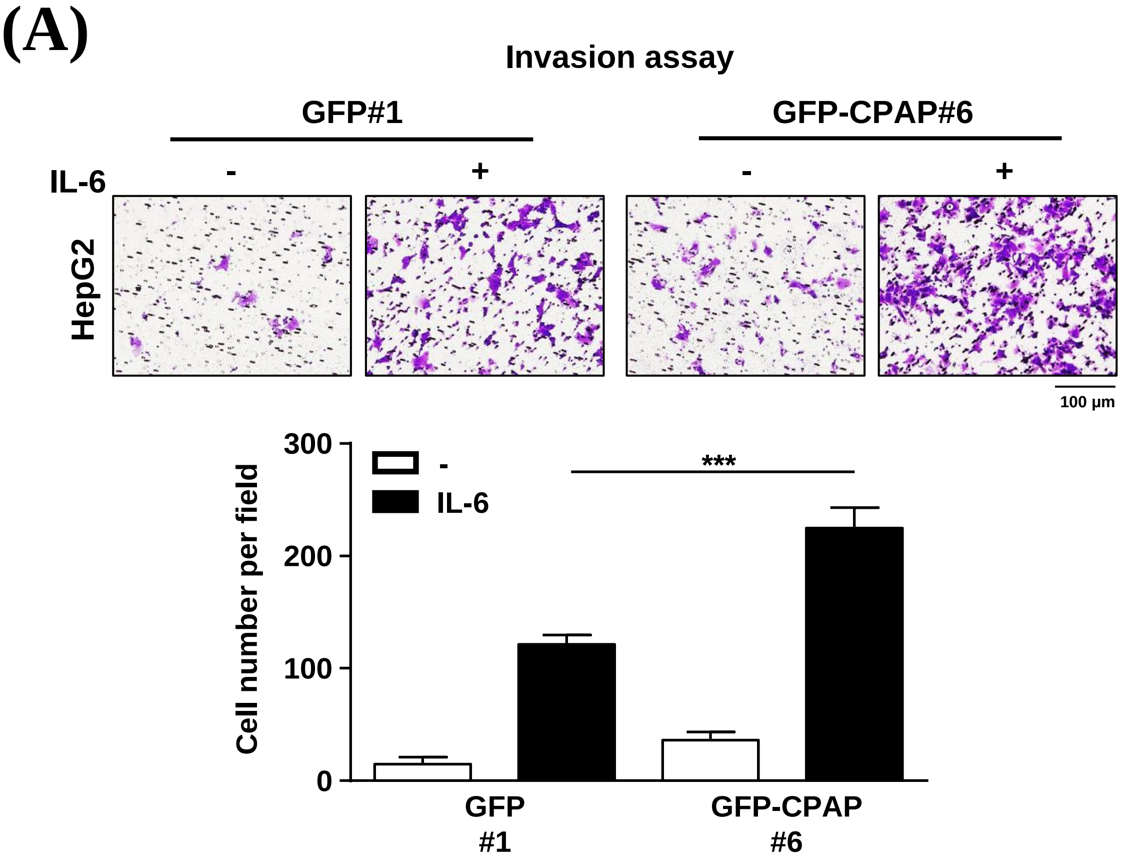

(B)

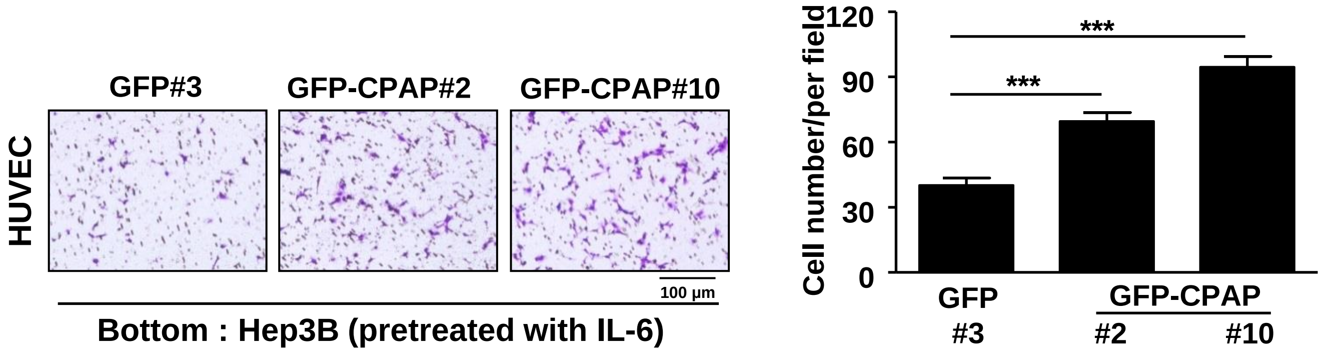

(C)

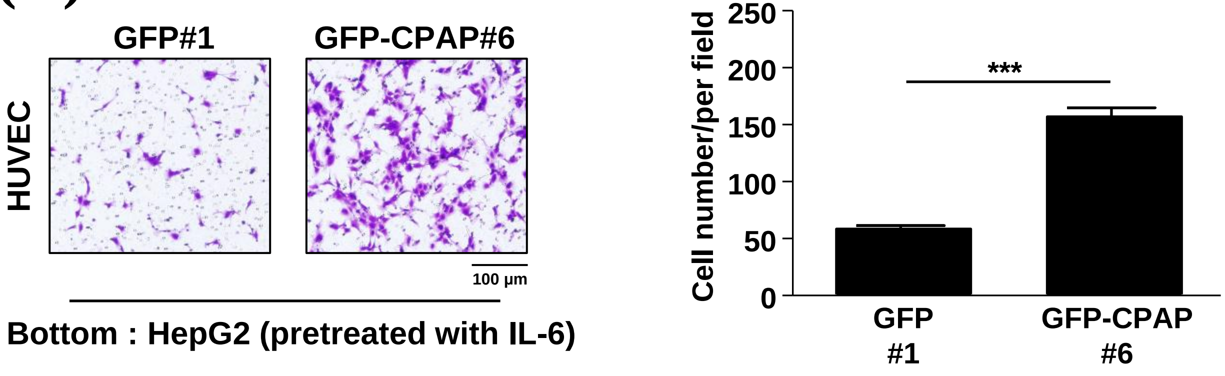

Supplementary Figure S11

(A)

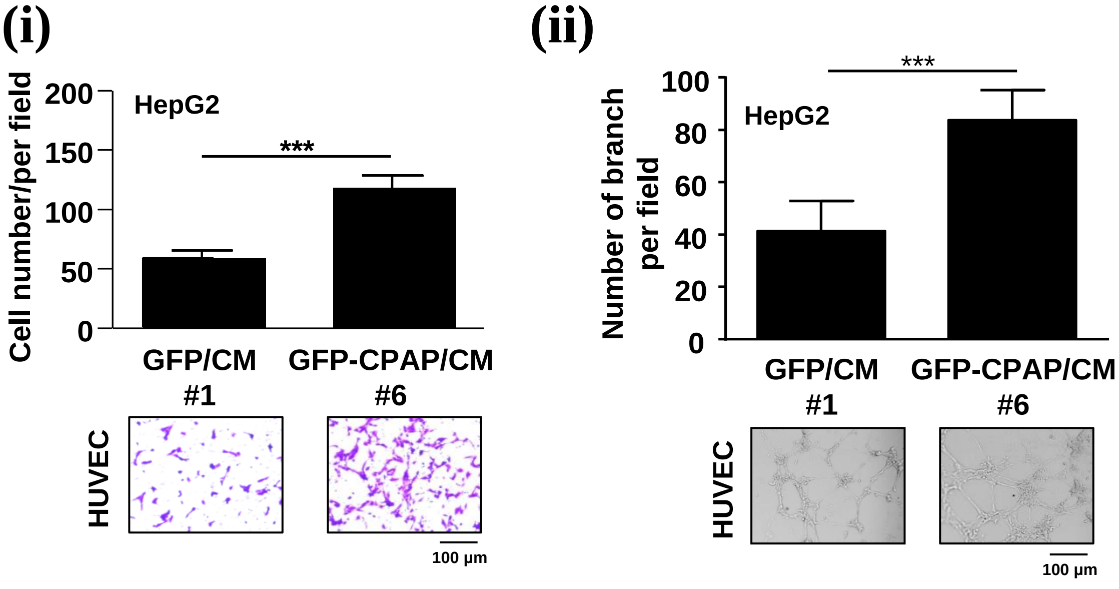

(B)

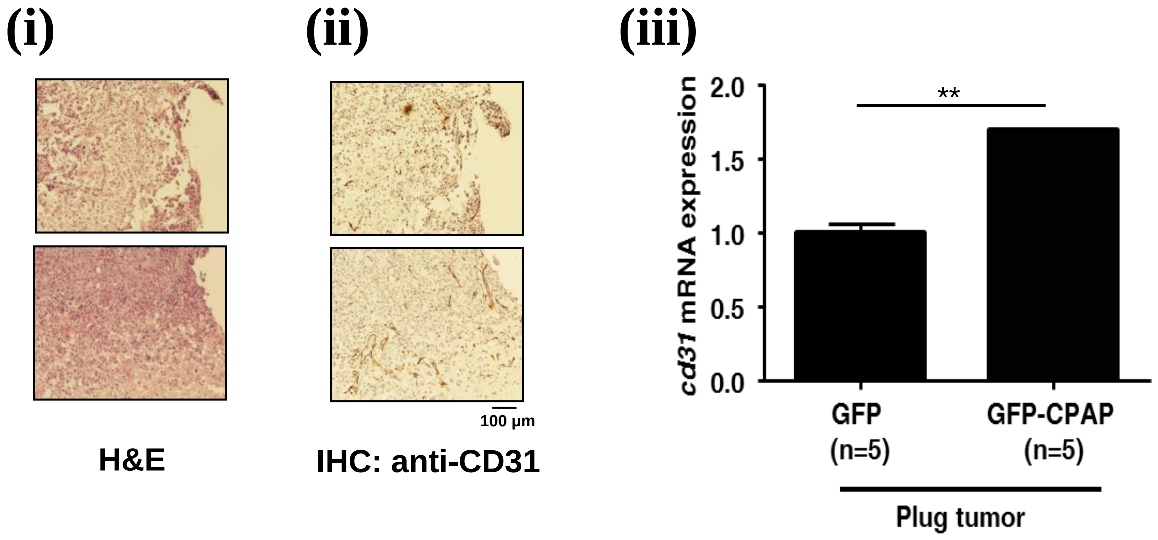

Supplementary Figure S12

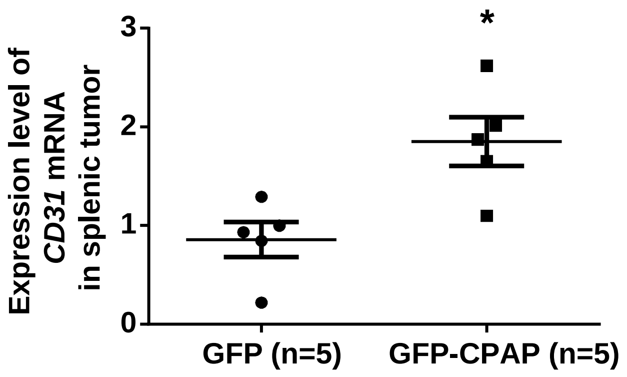

Supplementary Figure S13

(A)

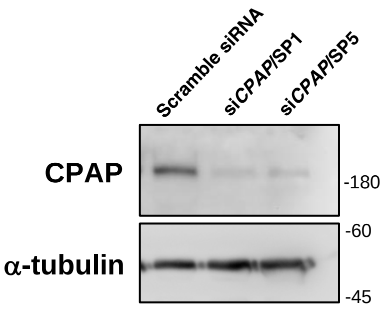

(B)

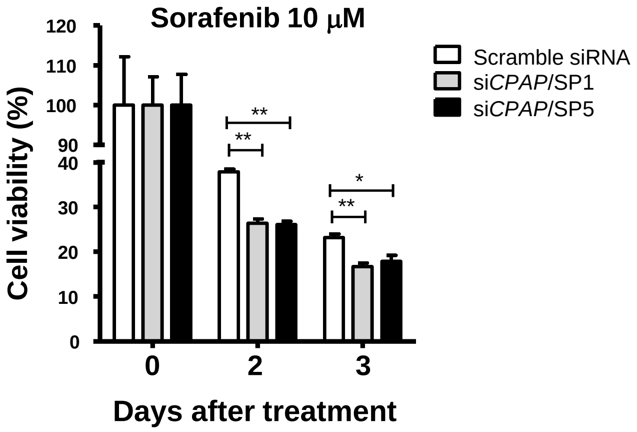

Supplementary Figure S14

(A)

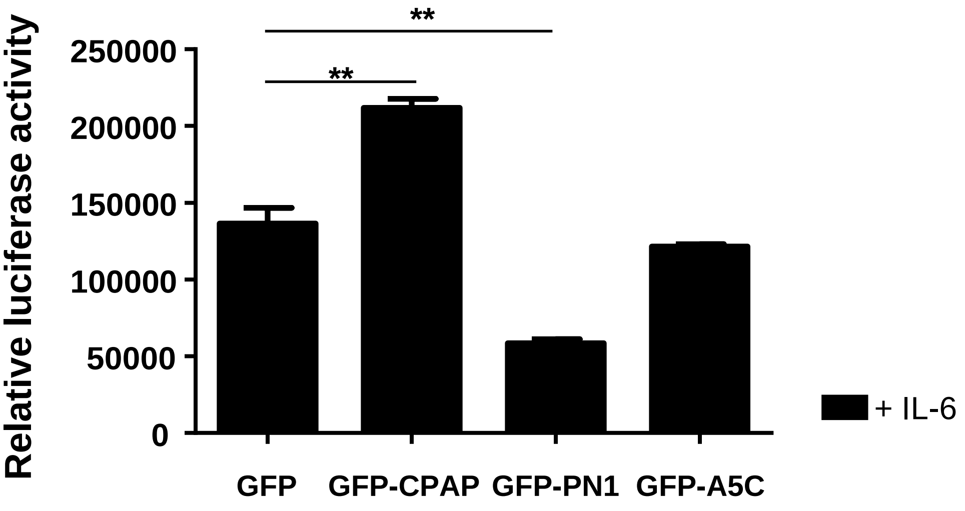

(B)

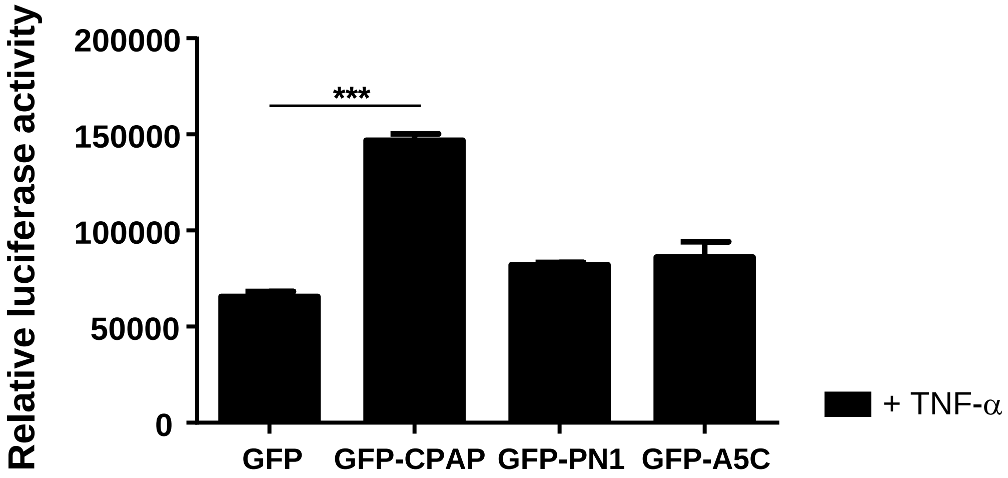

Supplementary Figure S15

(A)

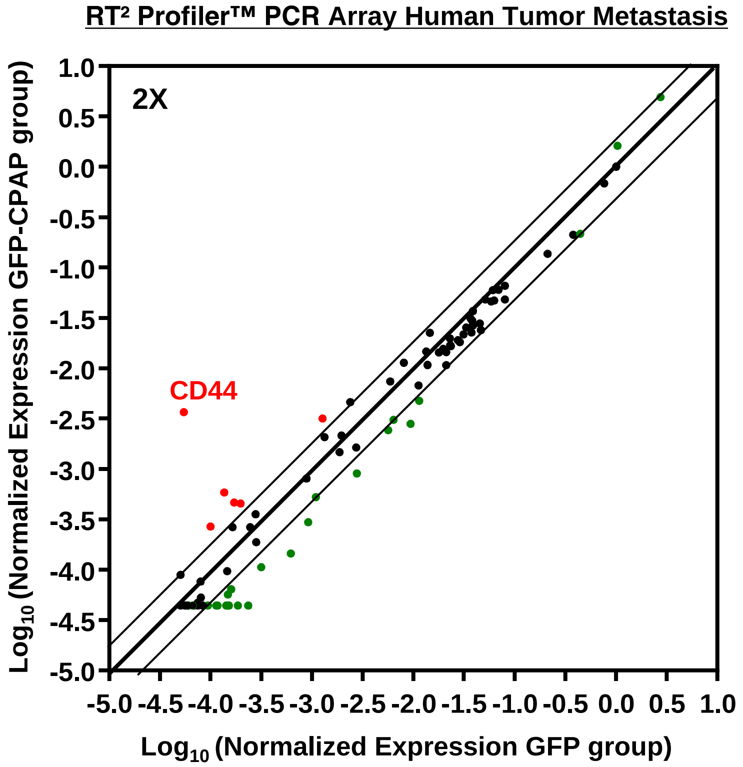

(B)

Genes Upregulated in GFP-CPAP vs. GFP			
**Gene Symbol**	**Description**	**Fold change**	**p-value**
CD44	CD44 molecule (Indian blood group)	67.3373	0.035
PLAUR	Plasminogen activator, urokinase receptor	4.2772	0.011
IL18	Interleukin 18 (interferon-gamma-inducing factor)	2.7195	0.296
MCAM	Melanoma cell adhesion molecule	2.6882	0.477
CDH1	Cadherin 1, type 1, E-cadherin (epithelial)	2.5082	0.041
HPSE	Heparanase	2.2974	0.658
