# Supplementary material for: CPAP promotes angiogenesis and metastasis by enhancing STAT3 activity
Source: Cell Death Differ. 2019 Sep 11;27(4):1259–73. doi: 10.1038/s41418-019-0413-7 (PMC7206147; doi:10.1038/s41418-019-0413-7)
Supplement: Supplementary file 2 — Supplementary Figures Legends [file 41418_2019_413_MOESM2_ESM.docx]

**Supplementary figure legends**

**Supplementary figure S1.** Total cell lysates from Hep3B **(A)**, Huh7 **(B)** and HepG2 **(C)** cells stably expressed with GFP or GFP-CPAP were collected for Western blot analysis using anti-GFP antibodies. GAPDH was used as a loading control.

**Supplementary figure S2. GFP-CPAP promotes metastasis.** Spleen tumors from GFP/Hep3B-injected mice without (n=2) or with (n=2) lung metastasis (from Figure 1E) were collected to analyze the expression of *CPAP* mRNA by RT-qPCR.

**Supplementary figure S3.** Hep3B cells were transfected with pSUPER-SP1 or pSUPER-SP5 to knock down the expression of endogenous CPAP (Cho et al., 2006; Yang et al., 2013). The levels of *CPAP* mRNA and CPAP protein were determined by RT-qPCR (upper) and Western blot analysis (lower), respectively. pSUPER-NS2 is a negative control for CPAP knocked-down. *CPAP* mRNA is normalized by *actin* mRNA; α-tubulin is a loading control for Western blot analysis. Student’s t-test, ***, *p＜*0.001.

**Supplementary figure S4. (A)** Phase-contrast microscopy showed a morphology change in GFP-CPAP/Hep3B stable cells (#2 and #10) compared with GFP/Hep3B stable cells (#3) or Hep3B parental cells. Arrows indicate the filipodia in GFP-CPAP/Hep3B cells. **(B)** GFP/Hep3B (#3) and GFP-CPAP/Hep3B (#10) cells were stained with DAPI, a DAN specific dye, and then subjected to immunofluorescence microscopy (DAPI) and phase-contrast microscopy (Phase) observation.

**Supplementary figure S5. Effect of AG490 in IL-6/STAT3 pathway.** After serum starvation, Huh7 cells were pretreated with (+) or without (-) 200 μM AG490 for 30 min and followed by treating with 25 ng/ml IL-6 for 6 h to determine the STAT3 transcriptional activity by a STAT3-driven luciferase reporter assay **(A)**, or treating with 25 ng/ml IL-6 for 10 min to determine the STAT3 transcriptional activity by Western blot analysis **(B)**. Student’s t-test, ***, *p＜*0.001.

**Supplementary figure S6.** The mRNA level of STAT3 target genes, *IL-8*, *VEGF*, *HIF-1α*, *IL-6*, or *ICAM-1* in CPAP knocked-down Hep3B cells was determined by RT-qPCR. Cells were cultured in growth medium **(A)**, or serum starved then treated with (■) or without (□) IL-6 for 24 h **(B)**. Student’s t-test, **, *p＜*0.01; *, *p＜*0.05.

**Supplementary figure S7. GFP-CPAP can form a complex with STAT3. (A)** Cells transfected with GFP-CPAP were starved, and then treated with 25 ng/ml IL-6 for 4 min. Total cell lysates were collected for immunoprecipitation (IP) assay using normal mouse IgG (IgG) or anti-STAT3 antibodies. The immunoprecipitated complexes were analyzed by Western blot analysis using anti-STAT3 or anti-GFP antibodies. **(B)** Cells transfected with GFP-CPAP and Myc-STAT3 were starved; IP assay was then performed as described above using normal mouse IgG (IgG) or anti-Myc antibodies, and subsequently analyzed by Western blot analysis using anti-Myc or anti-GFP antibodies. Red arrows indicate GFP-CPAP, STAT3 (A) or Myc-STAT3 (B). Stars are the IgG heavy chain.

**Supplementary figure S8. Mapping the interacting domains between CPAP and STAT3. (A)** Schematic diagram of CPAP cDNA truncated fragments. **(B)** Schematic diagram of STAT3 cDNA truncated fragments. **(C)** Huh7 cells transfected with GFP-CPAP truncated fragments were treated with IL-6 for 4 min. The interaction between GFP-CPAP truncated fragments and endogenous STAT3 was detected by *in situ* PLA using anti-GFP and anti-STAT3 antibodies, or by co-immunoprecipitation assay using anti-STAT3 antibodies. Lane1: GFP-PN1; lane2: GFP-CM; lane3: GFP-A5M2; lane 4: GFP-A5C. **(D)** Huh7 cells transfected with Myc-STAT3 truncated fragments were treated with IL-6 for 4 min. The interaction between endogenous CPAP and Myc-STAT3 truncated fragments was detected by *in situ* PLA using anti-CPAP and anti-myc antibodies or by co-immunoprecipitation assay using anti-CPAP antibodies. Lane1: Myc-STAT3/N; lane2: Myc-STAT3/C1; lane3: Myc-STAT3/C2. The red signals indicated the interaction between two proteins in the *in situ* PLA, and the quantitative results of PLA red signals are shown.

**Supplementary figure S9.** **CPAP and STAT3 can form a complex and co-localize to the nucleus upon IL-6 treatment.** **(A)** Cells with GFP or GFP-CPAP overexpression were starved and then treated with IL-6. Cytosolic and nuclear fractions were purified for Western blot analysis using antibodies as indicated. Tubulin is the cytosolic protein control; lamin A/C is the nuclear protein control. **(B)** Nuclear fractions from IL-6-treated cells were collected for immunoprecipitation assay using anti-STAT3 antibody, and the interacting GFP-CPAP in the immunoprecipitated complex was detected by anti-GFP antibody.

**Supplementary figure S10. CPAP promotes HCC invasion and HUVEC migration. (A)** GFP/HepG2 or GFP-CPAP/HepG2 stable cells treated with (+) or without (-) IL-6 were used to perform the transwell invasion assay. Quantitative results of invasive cells are shown. **(B-C)** HUVECs were seeded onto the Transwell insert, and the lower chamber was cultured with GFP or GFP-CPAP stably expressed Hep3B (B) or HepG2 (C) cells. Quantitative results of migrated HUVECs are shown. Student’s t-test, ***, *p* < 0.001.

**Supplementary figure S11. (A)** Conditioned medium (CM) of HepG2 stable cells was collected to perform HUVEC Transwell migration assay (i) and tube formation assay (ii). The quantitative results are shown. **(B)** The excised Matrigel plugs (from Figure 5D-i) were collected to perform H&E stain (i) and an IHC analysis using the anti-CD31 antibody (ii). The expression level of *CD31* mRNA in plug tumors was determined by TaqMan qPCR (iii). Student’s t-test, ***, *p* < 0.001; **, *p* < 0.01.

**Supplementary figure S12.** Expression level of *CD31* mRNA in GFP or GFP-CPAP-derived spleen tumors (from Figure 1E) was determined by TaqMan qPCR. N=5 for each groups. Student’s t-test, *, *p* < 0.05.

**Supplementary figure S13.** Hep3B cells were transfected with *CPAP* siRNA (siCPAP/SP1 or siCPAP/SP5) **(A)** and then treated with 10 μM of sorafenib by the indicated number of days. Cell viability was determined by counting cells using a hemocytometer (B). Student’s t-test, **, *p* < 0.01; *, *p* < 0.05.

**Supplementary figure S14. Analyzing the effects of CPAP truncated fragments in activating STAT3 or NF-κB.** Huh7 cells were co-transfected with GFP-CPAP truncated fragments (please see Supplementary Figure S8A) and STAT3-driven luciferase **(A)** or NF-κB-driven luciferase **(B).** After serum starved, the cells were treated with IL-6 (25 ng/ml) **(A)** or TNF-α (10 ng/ml) **(B)** for 24 h. The relative NF-κB or STAT3 transcription activity was determined as described above. Student’s t-test, ***, *p* < 0.001; **, *p* < 0.01.

**Supplementary figure S15. GFP-CPAP can increase metastatic related genes expression.** Total RNA purified from IL-6-treated GFP/Hep3B or GFP-CPAP/Hep3B stable cells was subjected to analysis of the gene expression profile using the human tumor metastasis RT^2^ Profiler™ PCR array. Red dots show the more than two-fold upregulated genes and green dots are the two-fold downregulated genes in GFP-CPAP/Hep3B cells **(A)**. **(B)** The expression of genes in IL-6-treated GFP-CPAP-overexpressing cells versus IL-6-treated GFP-expressing cells is shown as fold change. Only genes of upregulated greater than two-fold are shown.
